# Toxicity‐dependent feasibility bounds for the escalation with overdose control approach in phase I cancer trials

**DOI:** 10.1002/sim.7280

**Published:** 2017-03-15

**Authors:** Graham M. Wheeler, Michael J. Sweeting, Adrian P. Mander

**Affiliations:** ^1^ Cancer Research UK and UCL Cancer Trials CentreUniversity College LondonU.K.; ^2^ MRC Biostatistics Unit Hub for Trials Methodology ResearchCambridge Institute of Public HealthCambridgeU.K.; ^3^ Cardiovascular Epidemiology UnitStrangeways Research Laboratory University of CambridgeU.K.

**Keywords:** dose‐escalation, Bayesian adaptive designs, maximum tolerated dose, phase I trials

## Abstract

Phase I trials of anti‐cancer therapies aim to identify a maximum tolerated dose (MTD), defined as the dose that causes unacceptable toxicity in a target proportion of patients. Both rule‐based and model‐based methods have been proposed for MTD recommendation. The escalation with overdose control (EWOC) approach is a model‐based design where the dose assigned to the next patient is one that, given all available data, has a posterior probability of exceeding the MTD equal to a pre‐specified value known as the feasibility bound. The aim is to conservatively dose‐escalate and approach the MTD, avoiding severe overdosing early on in a trial. The EWOC approach has been applied in practice with the feasibility bound either fixed or varying throughout a trial, yet some of the methods may recommend incoherent dose‐escalation, that is, an increase in dose after observing severe toxicity at the current dose. We present examples where varying feasibility bounds have been used in practice, and propose a toxicity‐dependent feasibility bound approach that guarantees coherent dose‐escalation and incorporates the desirable features of other EWOC approaches. We show via detailed simulation studies that the toxicity‐dependent feasibility bound approach provides improved MTD recommendation properties to the original EWOC approach for both discrete and continuous doses across most dose‐toxicity scenarios, with comparable performance to other approaches without recommending incoherent dose escalation. © 2017 The Authors. Statistics in Medicine Published by John Wiley & Sons Ltd.

## 1 Introduction

Phase I clinical trials mark the first experimentation of a new drug in a human population. For cytotoxic anti‐cancer drugs, the aim of a phase I trial is to gradually adapt the dose level of the drug given to patients in order to identify the Maximum Tolerated Dose (MTD) of the experimental treatment. Various definitions for the MTD exist 1, but it is commonly regarded as the largest dose that leads to unacceptable toxicity in a target proportion, *θ*, of patients 2. The rationale for targeting such a dose is based on the assumption that higher doses will be more effective, yet more toxic 3, and that toxicity is tolerable for optimal anti‐tumour activity 4. Commonly, toxicity frequency and severity data are reduced to a single binary outcome, which denotes whether a dose‐limiting toxicity (DLT) has occurred or not 1. Therefore, for a pre‐specified target toxicity level (TTL) of *θ*, the definition of the MTD can be expressed mathematically as

(1) $$P\left(\text{DLT}\;|\;\text{dose}=\text{MTD}\right)=\theta .$$

The traditional approach for performing phase I dose‐escalation studies has long been the 3+3 design 5, 6, which remains popular in practice 1, 7, 8, despite having several pitfalls including slow dose‐escalation, not using all trial data to make dose‐escalation decisions, not having a pre‐determined target, poor MTD identification properties and no statistical justification 9, 10, 11, 12, 13, 14, 15. In order to overcome such pitfalls, several novel model‐based designs have been proposed. The Escalation with Overdose Control (EWOC) approach 4 is a Bayesian adaptive design that aims to reduce the risk of overdosing patients by choosing doses with a posterior probability of being above the true MTD equal to some value known as the *feasibility bound*. The feasibility bound, denoted as *α*, controls how conservative dose‐escalation is and was originally suggested to be a fixed constant throughout the trial. Several publications 16, 17, 18 describe trials where *α* increases during the trial so that eventually dose selection is based on the posterior median of the MTD distribution. Whilst such a design provides improved operating characteristics relative to the original EWOC approach with a fixed feasibility bound 19, there is no guarantee of dose‐escalation that is *coherent* 20, 21, 22; that is, dose escalation may be recommended despite having observed a DLT in the previous patient 23.

In this paper, we describe and investigate an approach for increasing the feasibility bound during a trial using the EWOC approach that guarantees coherent dose‐escalation behaviour and has comparable operating characteristics to other methods that have been implemented in practice. Section 2 describes the EWOC dose‐escalation approach, previously used adaptive feasibility bounds and outlines the proposed approach, which is dependent on the number of non‐DLT responses observed in the trial; we refer to this as the toxicity‐dependent feasibility bound (TDFB) approach. In Section 3, we describe a comprehensive simulation study comparing the TDFB approach to other EWOC‐based approaches, and Section 4 presents the results of this study and sensitivity analyses assuming misspecification of the dose‐toxicity model. We conclude with a discussion of the findings and limitations of our approach in Section 5 and offer recommendations for future trials.

## 2 Methods

### 2.1 Escalation with overdose control

Let *Y*_*i*_ be a binary random variable such that *Y*_*i*_=1 if patient *i* experiences a DLT and *Y*_*i*_=0 otherwise. For a dose range bounded below by $x_{\min}$ and above by $x_{\max}$, denote the probability of DLT for patient *i* at dose level $x\in \left[x_{\min},x_{\max}\right]$ by $\pi \left(x;\beta \right)$, where ***β*** is a parameter vector. Several structural forms for $\pi \left(x;\beta \right)$ have been proposed 21, but we shall only consider the two‐parameter logistic model proposed in the original EWOC paper 4, that is,

(2) $$P\left(Y_{i}=1\;|\;\text{dose}=x\right)=\pi \left(x;\beta_{0},\beta_{1}\right)=\frac{\exp\left(\beta_{0}+\beta_{1}x\right)}{1+\exp\left(\beta_{0}+\beta_{1}x\right)},$$

where *β*_0_ and *β*_1_ are parameters to be estimated, with the assumption that *β*_1_>0 to ensure the assumption of monotonicity is satisfied. We may rearrange equation (2) using equation (1) to show that the MTD, denoted as *γ*, can be written as

(3) $$\gamma =\frac{\text{logit}\left(\theta \right)-\beta_{0}}{\beta_{1}}.$$

Under the original EWOC approach 4, the form of $\pi \left(x;\beta_{0},\beta_{1}\right)$ may be expressed in terms of two clinically relevant and interpretable parameters: the MTD *γ* as defined in equation (3); and the probability of DLT at the lowest dose level to be used in the trial, denoted as *ρ*_0_, that is,

(4) $$\rho_{0}=\pi \left(x_{\min};\beta_{0},\beta_{1}\right)=\frac{\exp\left(\beta_{0}+\beta_{1}x_{\min}\right)}{1+\exp\left(\beta_{0}+\beta_{1}x_{\min}\right)}.$$

These parameters are more meaningful to clinicians and can be used in the Bayesian updating procedure by placing prior distributions on *γ* and *ρ*_0_ 4, 24. Babb *et al.* 4 suggest a Uniform prior distribution for *γ* over the interval $\left[x_{\min},x_{\max}\right]$, and a Uniform prior distribution for *ρ*_0_ over the interval $\left[0,\theta \right]$, since *ρ*_0_>*θ* implies that the MTD *γ* is lower than $x_{\min}$. Alternative prior distributions for *γ* and *ρ*_0_ have been investigated, which either induce a particular correlation structure between *γ* and *ρ*_0_, or do not truncate the domain of *γ* from above 25.

Subsequent calculations are conditional on the event *Y*_1_=0, that is, the first patient did not experience a DLT. If *Y*_1_=1, it is recommended that the trial is suspended for safety concerns and the dose range re‐evaluated, or the trial is terminated 4, 18, 25. Given observed trial data $D_{n}=\left\{\left(x(i),y_{i}\right):\;i=1,\ldots ,n\right\}$, where patient i∈{1,…,n} received dose $x(i)\in \left[x_{\min},x_{\max}\right]$ and had outcome *y*_*i*_ recorded, and a joint prior distribution $f\left(\gamma ,\rho_{0}\right)$ for parameters *γ* and *ρ*_0_, the marginal posterior cumulative distribution function for the MTD is

(5) $$\begin{array}{ccc} H_{n}(\gamma^{\prime }) & =P(\gamma ⩽\gamma^{\prime }\;|\;D_{n})=\int_{x_{\min}}^{\gamma^{\prime }}\int_{0}^{\theta }g(\gamma ,\rho_{0}\;|\;D_{n})\;d\rho_{0}\;d\gamma & \\ & =\int_{x_{\min}}^{\gamma^{\prime }}\int_{0}^{\theta }\frac{L(\gamma ,\rho_{0}\;|\;D_{n})f(\gamma ,\rho_{0})}{\int_{x_{\min}}^{x_{\max}}\int_{0}^{\theta }L(\gamma ,\rho_{0}\;|\;D_{n})f(\gamma ,\rho_{0})\;d\rho_{0}\;d\gamma }\;d\rho_{0}\;d\gamma , \end{array}$$

where $L(\gamma ,\rho_{0}\;|\;D_{n})=\prod_{i=1}^{n}\pi {\left(x;\gamma ,\rho_{0}\right)}^{y_{i}}{\left(1-\pi (x;\gamma ,\rho_{0})\right)}^{1-y_{i}}$ is the likelihood and $g(\gamma ,\rho_{0}\;|\;D_{n})$ is the joint posterior distribution function for *γ* and *ρ*_0_.

Under the EWOC approach, dose allocation for the next cohort of patients is determined by selecting a specified percentile from the posterior MTD distribution. The percentile of choice, known as the feasibility bound and denoted as *α*, governs the degree of conservatism present in the trial. Assuming a cohort size of one patient, the dose for the ${\left(n+1\right)}^{th}$ patient is

(6) $$x\left(n+1\right)=H_{n}^{-1}\left(\alpha \right).$$

The feasibility bound can be interpreted via a decision‐theoretic loss function as the relative preference of underdosing a patient compared to overdosing a patient. For any *δ*>0, the loss incurred by overdosing a patient (with respect to the MTD *γ*) by *δ* units is $\frac{1-\alpha }{\alpha }$ times greater than underdosing a patient by *δ* units 4, 16.

Different proposals have been made for choosing an MTD at the end of the trial. Babb *et al.* 4 suggest the recommended MTD should be the posterior mean of the MTD distribution, although in clinical practice, it is often the case that the MTD is the dose that would be given if a new patient were recruited into the trial. Berry *et al.* 26 estimate the MTD by a central estimate of the posterior MTD distribution (either mean, median or mode), and in a time‐to‐event adaptation of the EWOC approach, Tighiouart *et al.* 27 select the posterior median of the MTD distribution at the end of the trial.

### 2.2 Varying the feasibility bound

Under the EWOC approach with fixed *α* and uniform prior on *γ*, the dose for the second patient is the 100*α*th percentile of the dose range $\left[x_{\min},x_{\max}\right]$. Clinicians may consider this dose recommendation too high to administer to a patient early on in the trial 28 and prefer to conservatively escalate the dose at first, and then gradually relax the overdose control as more data are accrued. This combines the approaches of escalating as quickly as possible to the MTD, whilst simultaneously retaining some degree of overdose control. The idea of changing the feasibility bound during the trial has been discussed 2, 16, 19 and used in practice 16, 17, 18. At the start of the trial, *α* is set to some minimal level strictly less than 0.50, so that the first patients are treated at safe doses. As data accrue and the precision of the MTD distribution increases, *α* is gradually increased towards 0.50, at which point all future patients will be treated at the posterior median estimate of the MTD distribution. Allowing *α* to increase towards 0.50 also overcomes a concern raised by Berry *et al.* 26 that when *α* is fixed at some level below 0.50, the MTD estimated using the mean or median of *γ* may be much larger than any dose given to patients during the trial.

Chu *et al.* 19 proposed a varying feasibility bound approach, which they called the *Hybrid design*; for a trial of fixed sample size *N*,*α* is initially set at some minimum, denoted $\alpha_{\min}$, and increased in a stepwise manner so that it reaches 0.50 after patient *N*/2. They showed via simulation studies that the Hybrid design gave comparable operating characteristics to the modified Continual Reassessment Method (CRM) 29, 30, and both of these approaches were better at identifying the correct MTD relative to the conventional EWOC approach with fixed *α*.

However, whilst increasing the feasibility bound in a step‐wise procedure can provide a desirable blend between conservative dose‐escalation at the start of a trial with reaching the true MTD quicker than the original EWOC approach, a problem still remains. If the feasibility bound is increased, despite the most recent patient experiencing a DLT, the recommendation may be to treat the next patient at a higher dose level. Investigators and clinicians would prefer to have a trial design that can guarantee *coherent* dose‐escalation 20 as well as offer favourable experimentation and MTD recommendation properties. A trial design that is coherent will never recommend a dose higher than the previous patient's dose if the previous patient experienced a DLT, nor recommend a dose lower than the previous patient's dose if the previous patient did not experience a DLT 21. The unmodified CRM approach has been shown to be coherent 20, 21, 31, and the EWOC approach is coherent for n⩾2 18. However, if increases in *α* occur regardless of the DLT response of the preceding patients, an incoherent dose‐escalation, or *coherence violation*, may occur 23. Bartroff and Lai 22 considered coherence violations in their evaluation of the EWOC approach with a linearly increasing feasibility bound; their approach to prevent incoherence was to restrict the choice of dose level to be from either above or below the previous dose if the previous patient did not or did experience a DLT respectively.

### 2.3 A toxicity‐dependent feasibility bound

We now present an approach for varying the feasibility bound that guarantees coherent dose escalation. The method also provides less conservative escalation when no DLT is observed at the current patient compared to a linearly increasing feasibility bound, such as that used in the Hybrid design. The TDFB to determine the dose given to patient *n*+1 is

(7) $$\alpha_{n+1}=\min\left\{0.50,\;\alpha_{\min}+\left(0.50-\alpha_{\min}\right)\frac{\left(n-1-\overset{n}{\underset{i=1}{\sum }}y_{i}\right)}{S}\right\},$$

where $\left(n-1-\sum_{i=1}^{n}y_{i}\right)$ is the number of patients not experiencing DLTs out of the *n*−1 patients dosed after patient 1 (since if *y*_1_=1, we stop the trial due to safety concerns) and *S* is a strictly positive constant chosen before the trial. This feasibility bound is non‐decreasing, has a value of $\alpha_{\min}$ when *n*=1 (i.e. $\alpha_{2}=\alpha_{\min}$) and does not exceed 0.50. The constant *S* determines the speed at which *α*_*n*_ increases with *n* and can be interpreted as the number of non‐DLT responses one must observe (excluding the first patient) before the posterior median of the MTD distribution can be used for dose selection. To choose a sensible value for *S*, consider the expectation of *α*_*n*+1_ and the patient number for which *α*_*n*+1_ is expected to first reach 0.50. Ignoring the minimum aspect of equation (7), the expectation of *α*_*n*+1_ is

(8) $$\begin{array}{ccc} E\left(\alpha_{n+1}\right) & =E\left(\alpha_{\min}+\left(0.50-\alpha_{\min}\right)\frac{\left(n-1-\overset{n}{\underset{i=1}{\sum }}Y_{i}\right)}{S}\right) & \\ & =\alpha_{\min}+\left(0.50-\alpha_{\min}\right)\frac{\left(n-1-E\left(\overset{n}{\underset{i=1}{\sum }}Y_{i}\right)\right)}{S}. \end{array}$$

Since we are choosing a value of *S* before the trial begins, the DLT outcomes of patients i=1,…,n are unobserved random variables *Y*_*i*_, rather than observed outcomes *y*_*i*_. In dose finding trials where the goal is to treat patients at the dose with probability of DLT equal to *θ*, the long‐run expectation of $\sum_{i=1}^{n}Y_{i}$ is (*n*−1)*θ*, that is, we expect on average (*n*−1)*θ* DLTs in (*n*−1) patients. Therefore, setting $E(\sum_{i=1}^{n}Y_{i})=(n-1)\theta$ gives

(9) $$E\left(\alpha_{n+1}\right)=\alpha_{\min}+\left(0.50-\alpha_{\min}\right)\left(\frac{(n-1)\left(1-\theta \right)}{S}\right).$$

One can use equation (9) to decide after how many patients, on average, the feasibility bound should reach 0.50. For example, if we wish the expectation of *α*_*n*+1_ to be 0.50 when half of *N* available patients have been dosed, we can set $n=\frac{N}{2}$ and $E\left(\alpha_{\frac{N}{2}+1}\right)=0.50$ so that

(10) $$\begin{array}{ccc} E\left(\alpha_{\frac{N}{2}+1}\right)=0.50 & =\alpha_{\min}+\left(0.50-\alpha_{\min}\right)\left(\frac{(\frac{N}{2}-1)\left(1-\theta \right)}{S}\right) & \\ \Rightarrow S & =\left(\frac{N}{2}-1\right)\left(1-\theta \right). \end{array}$$

Therefore, one may select *S* as a function of the total number of patients available, *N*, and the TTL *θ*. If, for example, we have *N*=40 patients and *θ*=1/3, then setting $S=12\frac{2}{3}$ will mean that on average, *α*_*n*_ will first reach 0.50 after 20 of the 40 patients have been observed. If we consider the difference in expectations of successive feasibility bounds, then

(11) $$E(\alpha_{n+2})-E(\alpha_{n+1})=\left(1-E(Y_{n+1})\right)\frac{0.50-\alpha_{\min}}{S}.$$

Setting $S=\left(\frac{N}{2}-1\right)(1-\theta )$, we have

(12) $$E(\alpha_{n+2})-E(\alpha_{n+1})=\frac{1-E(Y_{n+1})}{1-\theta }\frac{0.50-\alpha_{\min}}{\frac{N}{2}-1}.$$

Under the Hybrid design of Chu *et al.*, the rate of increase in successive feasibility bounds is $\frac{0.50-\alpha_{\min}}{N/2-1}$. Therefore, if $E(Y_{n+1})<\theta$, then the increase in expectation of the feasibility bound is greater than that of the Hybrid design. Similarly, if $E(Y_{n+1})>\theta$, the rate of increase in successive feasibility bounds is slower than the Hybrid design. These properties are sensible because we wish to be less conservative when the expected probability of DLT for the next patient is less than *θ*, and more conservative when the expected probability of DLT for the next patient is more than *θ*. Furthermore, conservative dose‐escalation at the start of the trial coupled with quicker escalation to the MTD are still achievable by increasing the feasibility bound in the absence of DLTs.

The TDFB approach, or any EWOC approach whereby the feasibility bound only increases when the last patient does not experience a DLT, guarantees coherent dose escalation/de‐escalation behaviour. The proof of this is a simple extension of that for the coherence of the EWOC approach with fixed *α* given by Tighiouart and Rogatko 18.

## 3 Simulation study

**Figure 1:**
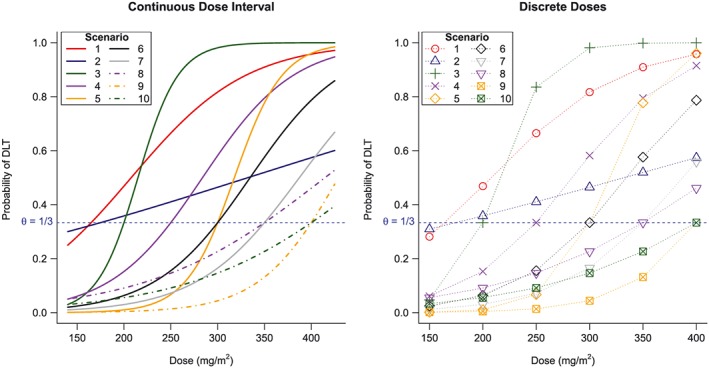
Dose‐toxicity scenarios for continuous dose interval (left plot) and discrete doses (right plot) used in simulation study. Target toxicity level θ=0.33 (blue dashed horizontal line). Lines of same colour have the same probability of dose‐limiting toxicity (DLT) at $x_{\min}$.

We describe and conduct a simulation study comparing several EWOC approaches with fixed and adaptive feasibility bounds. We consider the trial described by Babb *et al.* 4 that used the EWOC approach to find the MTD of 5‐fluorouracil (5‐FU) when given in combination with 20mg/m^2^ leucovorin and 0.5mg/m^2^ topotecan to patients with malignant solid tumours, with a TTL *θ* of $\frac{1}{3}$. We consider 10 different dose‐toxicity scenarios generated from a logistic model using true values for *γ* and *ρ*_0_ (Table 1), which gave steep, shallow and plateauing dose‐toxicity curves (Figure 1). Several scenarios have either the same *γ* or *ρ*_0_ value, so performance differences between scenarios can be assessed relative to these individual parameters. The EWOC approach has been used in practice to find the MTD over continuous dose intervals and discrete dose sets 28; therefore, for each scenario specified by *γ* and *ρ*_0_, we investigate sub‐scenarios with (i) a continuous dose interval, with dose selection made across the entire dose range of $L_{C}=\left[140,425\right]{\text{mg/m}}^{2}$ and rounded to the nearest integer, and (ii) six discrete dose levels $L_{D}=\{150,200,250,300,350,400\}\;{\text{mg/m}}^{2}$. For each scenario, we simulate 1000 trials of *N*=40 patients.

**Table 1:** Dose‐toxicity scenarios (for both continuous dose interval and discrete dose settings) used in simulation study. MTDs for discrete dose scenarios shown in bold.

Continuous dose scenarios							
Scenario	MTD (*γ*)	*ρ*_0_	$P\left(\text{DLT at}x_{\max}\right)$			*β*_0_	*β*_1_
1	165	0.25	0.97			−3.369	0.016
2	175	0.30	0.60			−1.464	0.004
3	200	0.03	1.00			−9.970	0.046
4	250	0.05	0.95			−5.810	0.020
5	300	0.001	0.98			−12.344	0.039
6	300	0.02	0.86			−6.691	0.020
7	350	0.01	0.67			−7.196	0.019
8	350	0.05	0.53			−4.445	0.011
9	400	0. 001	0.48			−10.253	0.024
10	400	0.03	0.40			−4.975	0.011

Discrete dose scenarios							
Scenario	P(DLT)						
	*d*_1_	*d*_2_		*d*_3_	*d*_4_	*d*_5_	*d*_6_
1	**0.28**	0.47		0.66	0.82	0.91	0.96
2	**0.31**	0.36		0.41	0.46	0.52	0.57
3	0.05	**0.33**		0.84	0.98	1.00	1.00
4	0.06	0.15		**0.33**	0.58	0.79	0.92
5	0.00	0.01		0.07	**0.33**	0.78	0.96
6	0.02	0.06		0.16	**0.33**	0.58	0.79
7	0.01	0.03		0.07	0.16	**0.33**	0.56
8	0.06	0.09		0.15	0.23	**0.33**	0.46
9	0.00	0.00		0.01	0.04	0.13	**0.33**
10	0.03	0.06		0.09	0.15	0.23	**0.33**

DLT, dose‐limiting toxicity; MTD, maximum tolerated dose.

We use a bivariate normal prior for the intercept and the log‐slope parameters *β*_0_ and $log(\beta_{1})$ 32; this facilitates the use of the R package bcrm 33, which we have tailored to include the adaptive feasibility bounds of interest. The main reason for using this package is to quickly conduct MCMC computations used to generate posterior inferences, which we detail in subsection 3.3. The bivariate normal prior used has parameters *μ*_0_=−2.56,*μ*_1_=−5.32,*σ*_0_=1.24,*σ*_1_=0.91 and *ρ*=−0.90 (see supplementary material for R code and plot). This corresponds to a prior mean (SD) of 0.20 (0.15) and 254 (477) for *ρ*_0_ and *γ*, respectively. At the end of the trial, we consider two estimators for the MTD; the posterior median of the MTD distribution $\hat{\gamma }=H_{N}^{-1}\left(0.50\right)$, and the dose that would be given to a new patient if they were recruited into the trial $\tilde{\gamma }=H_{N}^{-1}\left(\alpha_{N+1}\right)$. This is because we wish to not only compare the performance of all designs using the same MTD estimator but also acknowledge that in practice when *α* is fixed at a level well below 0.50, clinicians may be reluctant to use $\hat{\gamma }$ and therefore use the next recommended dose given the current trial data.

### 3.1 Approaches for comparison

We examine five approaches for dose selection using a feasibility bound *α*_*n*_:

1. EWOC – the original EWOC approach with *α*=0.25 for each trial;
2. TR design – the Tighiouart and Rogatko design 18, where $\alpha_{2}=\cdots =\alpha_{9}=0.25,\alpha_{n}=\alpha_{n-1}+0.05$ for n=10,…,14 and *α*_*n*_=0.50 for future patients;
3. Hybrid design – the feasibility bound begins at $\alpha_{\min}$ and increases in equal increments after each patient until $\alpha_{\frac{N}{2}+1}=\alpha_{21}=0.50$, then all future patients receive the posterior median of the MTD distribution;
4. Escalation in the absence of toxicity (EAT) design – the feasibility bound begins at $\alpha_{\min}=0.10$ and increases in increments of 0.05 only when the previous patient did not experience a DLT;
5. TDFB design – the feasibility bound begins at $\alpha_{\min}$ and increases as per equation (7) up to 0.50; for a trial with *N*=40 and $\theta =\frac{1}{3},S=12\frac{2}{3}$.

For the Hybrid and TDFB designs, we use both $\alpha_{\min}$ equal to 0.10 and 0.25.

### 3.2 Trial design

We outline the dose‐escalation method for the EWOC approach with a fixed or varying feasibility bound for a trial of *N* patients over dose set L, which may be the continuous interval $L_{C}$ or the discrete dose set $L_{D}$.

1. At the start of the trial, dose patient 1 at the lowest dose level $x_{\min}$. If *Y*_1_=1, stop the trial. Otherwise, proceed to (2).
2. For 1⩽n<N,
  - Set *α*_*n*+1_ using the feasibility bound method of choice.
  - With joint prior distribution *f*(*γ*,*ρ*_0_) and likelihood $L(\gamma ,\rho_{0}\;|\;D_{n})$, obtain posterior distribution $g(\gamma ,\rho_{0}\;|\;D_{n})$ and marginal cumulative distribution function *H*_*n*_(*γ*).
  - Treat patient *n*+1 at *x*(*n*+1) such that
    (13) $$x(n+1)={\text{arg min}}_{l\in L}|H_{n}^{-1}\left(\alpha_{n+1}\right)-l|,$$
    where *α*_*n*+1_ is the feasibility bound. This may be equal to some fixed *α* for all *n*(original EWOC approach), or non‐decreasing with *n*(TR design, Hybrid design, EAT design and TDFB design).
  - Observe *Y*_*n*+1_. Set *n*=*n*+1.
  - Repeat steps (a) – (d).
3. If *n*=*N*, stop the trial. Obtain *H*_*N*_(*γ*) and MTD estimate $\bar{\gamma }$ (either posterior median $\hat{\gamma }$ or next recommended dose $\tilde{\gamma }$).

### 3.3 Simulation set‐up

We simulated 1000 trials for each approach and dose‐toxicity scenario using the R package bcrm 33. Posterior distributions of *β*_0_ and *β*_1_ were updated by MCMC methods in JAGS 34 (called from bcrm), which were then used to generate the distributions of *γ* and *ρ*_0_. Two Markov chains were run with a burn‐in period of 20 000 iterations, followed by a posterior sample of 20 000 iterations, thinned at every two iterations. This was adequate for convergence of the Markov chains and for minimal autocorrelation of the posterior samples. We report the distributions of patient experimentation and final MTD recommendations, and also the mean bias and root mean squared error (RMSE) of the MTD recommendations. The mean bias and RMSE are respectively defined as

(14) $$\text{Mean Bias}=\frac{1}{1000}\overset{1000}{\underset{k=1}{\sum }}\left({\bar{\gamma }}_{k}-\gamma \right)\;\;\;\;\text{and}\;\;\;\;\;\text{RMSE}=\sqrt{\frac{1}{1000}\overset{1000}{\underset{k=1}{\sum }}{\left({\bar{\gamma }}_{k}-\gamma \right)}^{2}},$$

where *γ* is the true MTD and ${\bar{\gamma }}_{k}$ is the MTD recommendation (either the posterior median $\hat{\gamma }$ or the next recommended dose $\tilde{\gamma }$) at the end of the *k* ^*th*^ trial. We also summarise the accuracy of each approach in MTD recommendation using an accuracy index 21, such that the accuracy A of a dose‐escalation approach is defined as

(15) $$A=1-J\frac{\overset{J}{\underset{j=1}{\sum }}{\left(\pi (d_{j})-\theta \right)}^{2}p_{j}}{\overset{J}{\underset{j=1}{\sum }}{\left(\pi (d_{j})-\theta \right)}^{2}},$$

where *J* is the number of dose levels in the trial, *π*(*d*_*j*_) is the true probability of DLT at dose level *d*_*j*_ and *p*_*j*_ is the probability that dose level *d*_*j*_ is chosen as the MTD at the end of the trial. A maximum score of A=1 implies an approach selects the correct MTD all the time, with lower scores implying lower accuracy.

## 4 Simulation results

Table 2 shows the percentage of coherence violations (with standard errors) for approaches that do not guarantee coherent dose escalation (all approaches not listed guarantee coherence and therefore presented no violations). In the continuous dose scenarios, coherence violations were observed for the TR and Hybrid approaches in all scenarios (mean 3.5mg/m^2^, range 1−11mg/m^2^). The TR approach has a higher percentage of coherence violations across all scenarios, due to the larger increases in *α*_*n*_ between patients (TR = 0.05; Hybrid $(\alpha_{\min}=0.10)=0.40/19\approx 0.021$; Hybrid $(\alpha_{\min}=0.25)=0.25/19\approx 0.013$). For discrete dose scenarios, there are far fewer coherence violations (0.005 – 0.02%); much larger increases in *α*_*n*_ are required for an approach to recommend a dose escalation after observing a DLT. However, an incoherent dose escalation results in an increase in dose by 50mg/m^2^ after observing a DLT.

**Table 2:** Coherence violation percentages (%) with SE over 1000 trials during increases in feasibility bound (TR design = 5 per trial; Hybrid design = 20 per trial) for approaches that do not guarantee coherence (continuous and discrete dose scenarios).

		Continuous			Discrete	
Scenario	Approach	%	SE		%	SE
1	TR	0.240	0.006		0	0
	Hybrid ( $\alpha_{\min}=0.10$)	0.058	0.002		0.005	0.005
	Hybrid ( $\alpha_{\min}=0.25$)	0.074	0.002		0	0
2	TR	0.320	0.007		0	0
	Hybrid ( $\alpha_{\min}=0.10$)	0.032	0.001		0	0
	Hybrid ( $\alpha_{\min}=0.25$)	0.063	0.002		0.005	0.005
3	TR	0.260	0.006		0	0
	Hybrid ( $\alpha_{\min}=0.10$)	0.021	0.001		0	0
	Hybrid ( $\alpha_{\min}=0.25$)	0.037	0.001		0	0
4	TR	0.340	0.007		0	0
	Hybrid ( $\alpha_{\min}=0.10$)	0.042	0.001		0	0
	Hybrid ( $\alpha_{\min}=0.25$)	0.032	0.001		0	0
5	TR	0.120	0.005		0	0
	Hybrid ( $\alpha_{\min}=0.10$)	0.016	0.001		0	0
	Hybrid ( $\alpha_{\min}=0.25$)	0.032	0.001		0	0
6	TR	0.420	0.007		0	0
	Hybrid ( $\alpha_{\min}=0.10$)	0.032	0.001		0	0
	Hybrid ( $\alpha_{\min}=0.25$)	0.068	0.002		0	0
7	TR	0.240	0.006		0.020	0.002
	Hybrid ( $\alpha_{\min}=0.10$)	0.016	0.001		0	0
	Hybrid ( $\alpha_{\min}=0.25$)	0.037	0.001		0	0
8	TR	0.240	0.006		0	0
	Hybrid ( $\alpha_{\min}=0.10$)	0.026	0.001		0.005	0.005
	Hybrid ( $\alpha_{\min}=0.25$)	0.026	0.001		0	0
9	TR	0.120	0.005		0	0
	Hybrid ( $\alpha_{\min}=0.10$)	0.011	0.001		0	0
	Hybrid ( $\alpha_{\min}=0.25$)	0.032	0.001		0	0
10	TR	0.100	0.004		0	0
	Hybrid ( $\alpha_{\min}=0.10$)	0.032	0.001		0.005	0.005
	Hybrid ( $\alpha_{\min}=0.25$)	0.021	0.001		0	0

SE, standard errors; TR, Tighiouart and Rogatko design.

We now consider the performance of each approach across continuous and discrete scenarios, specifically analysing accuracy index scores, mean bias, RMSE and mean number of DLTs; detailed results tables are provided as supplementary material (Tables S1, S2, S3 and S4).

### 4.1 Continuous dose interval

**Figure 2:**
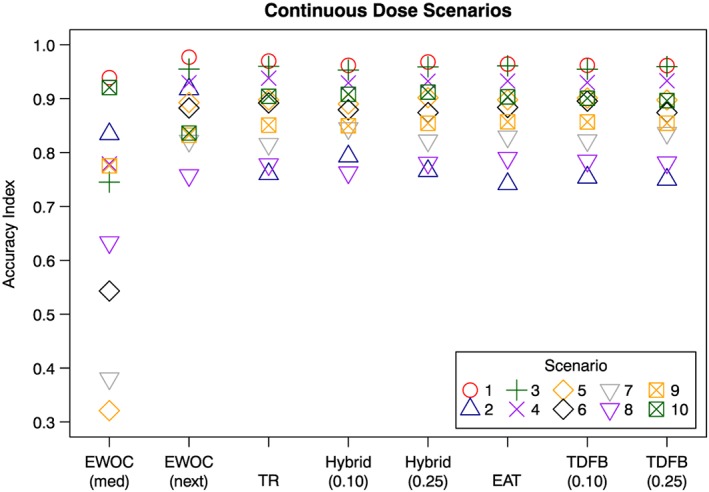
Accuracy index scores for each approach applied to continuous dose interval scenarios. EAT, escalation in the absence of toxicity; EWOC, escalation with overdose control; TDFB, toxicity‐dependent feasibility bound; TR, Tighiouart and Rogatko design.

**Figure 3:**
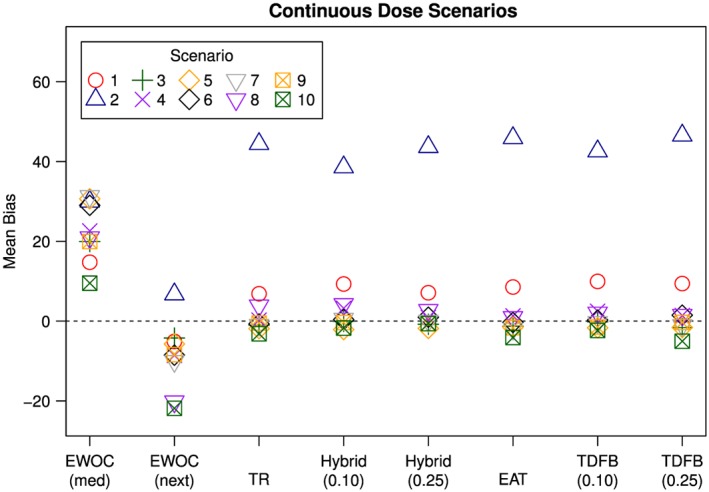
Mean bias of maximum tolerated dose recommendations per approach for continuous dose scenarios. Black dashed line indicates a mean bias of 0. EAT, escalation in the absence of toxicity; EWOC, escalation with overdose control; TDFB, toxicity‐dependent feasibility bound; TR, Tighiouart and Rogatko design.

**Figure 4:**
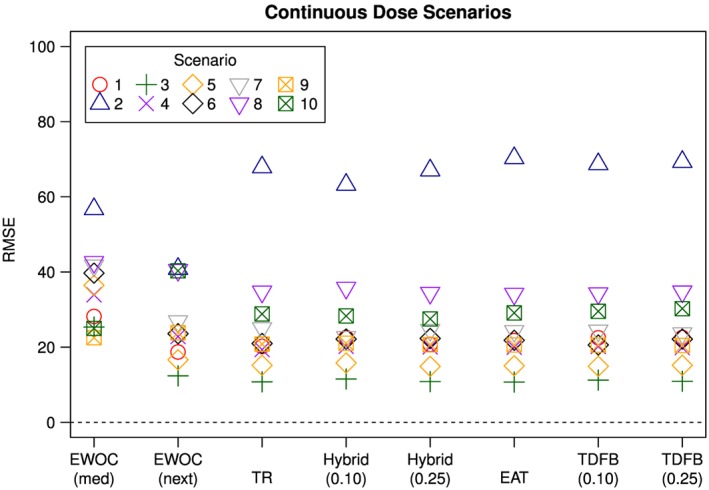
Root mean square error (RMSE) of maximum tolerated dose recommendations per approach for continuous dose scenarios. Black dashed line indicates an RMSE of 0. EAT, escalation in the absence of toxicity; EWOC, escalation with overdose control; TDFB, toxicity‐dependent feasibility bound; TR, Tighiouart and Rogatko design.

**Figure 5:**
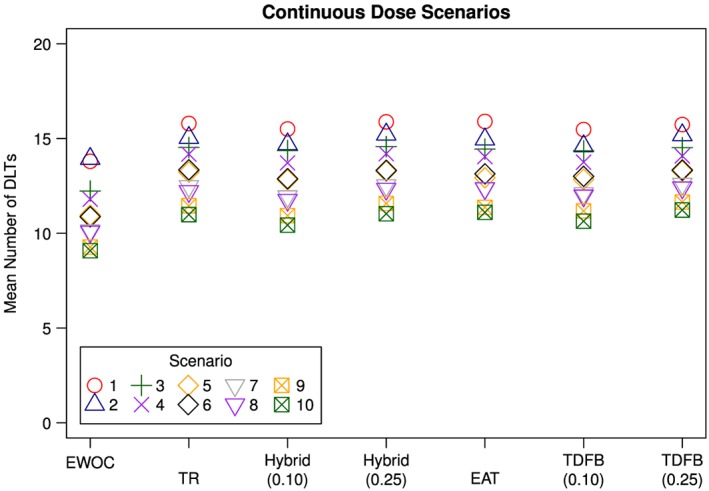
Mean number of dose‐limiting toxicity (DLTs) per approach for continuous dose scenarios. EAT, escalation in the absence of toxicity; EWOC, escalation with overdose control; TDFB, toxicity‐dependent feasibility bound; TR, Tighiouart and Rogatko design.

Figures 2, 3, 4, 5 show the performance of each approach across the continuous dose scenarios. Figure 2 shows the EWOC approach with a median MTD estimator has much lower accuracy across most scenarios than all other approaches, which typically score above 0.80. The remaining coherent approaches (EWOC with next dose MTD estimator, EAT and TDFB) have similar accuracy scores to those that do not guarantee coherence (TR and Hybrid approaches).

Figure 3 shows the mean bias around MTD estimates, indicating that the EWOC approach tends to lead to more biased results than approaches with escalating feasibility bounds; using the median MTD estimator biases estimates above the truth, whereas the next dose estimator biases estimates below the truth. Scenario 2 provides an exception to this trend; the gradual slope of the dose‐toxicity curve and high value of *ρ*_0_=0.30 mean that the most conservative escalation approaches (EWOC) perform better than those that escalate the feasibility bound. Figure 4 shows all approaches that increase feasibility bounds provide give lower RMSEs (except scenario 2) than the EWOC approach, and that performance differs little between TR, Hybrid, EAT and TDFB.

With respect to experimentation, Figure 5 shows fewer DLTs on average when using the EWOC approach than all other approaches; the Hybrid and TDFB ( $\alpha_{\min}=0.10$) approaches give slightly lower DLTs on average compared to TR, EAT and Hybrid and TDFB ( $\alpha_{\min}=0.25$). Furthermore, when increasing the feasibility bound, the choice of $\alpha_{\min}$ does not have much impact on the percentage of patients receiving doses with true probabilities of DLT in the [0.30,0.35) interval, though there is a noticeable difference in how much experimentation is conducted at the doses with the lowest DLT probabilities per scenario (Table S1). Approaches with $\alpha_{\min}=0.10$ (Hybrid ( $\alpha_{\min}=0.10$), EAT and TDFB ( $\alpha_{\min}=0.10$)) exhibit more experimentation at lower doses (all but scenario 3, where the dose‐toxicity curve is very steep) compared with approaches with $\alpha_{\min}=0.25$, which is expected because a lower $\alpha_{\min}$ is used.

### 4.2 Discrete dose levels

**Figure 6:**
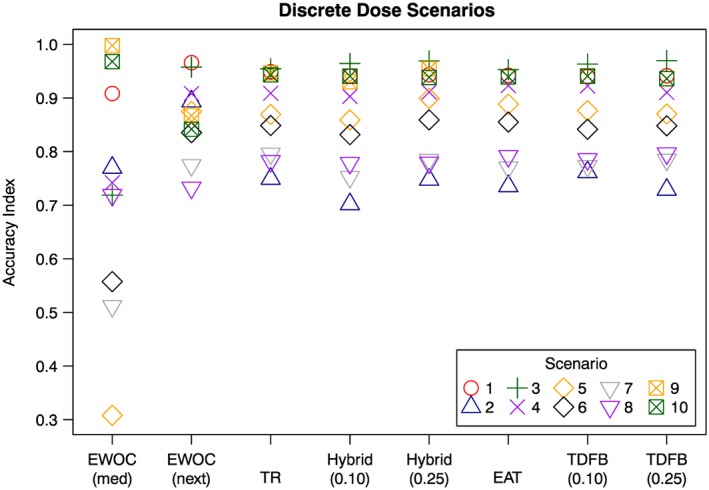
Accuracy index scores for each approach applied to discrete dose interval scenarios. EAT, escalation in the absence of toxicity; EWOC, escalation with overdose control; TDFB, toxicity‐dependent feasibility bound; TR, Tighiouart and Rogatko design.

**Figure 7:**
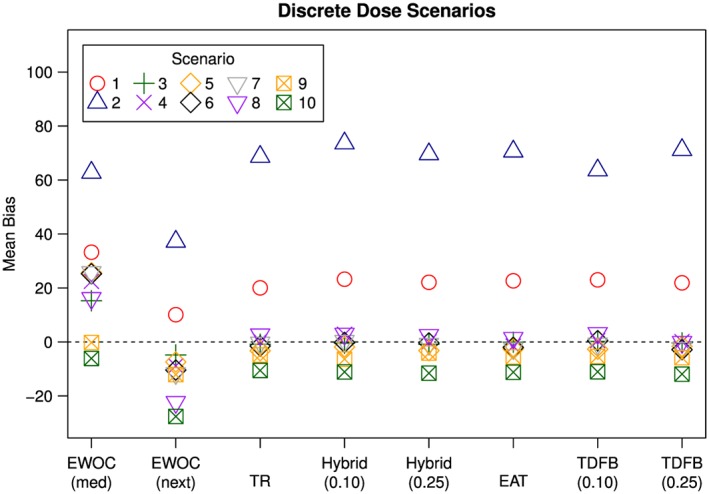
Mean bias of maximum tolerated dose recommendations per approach for discrete dose scenarios. Black dashed line indicates a mean bias of 0. EAT, escalation in the absence of toxicity; EWOC, escalation with overdose control; TDFB, toxicity‐dependent feasibility bound; TR, Tighiouart and Rogatko design.

**Figure 8:**
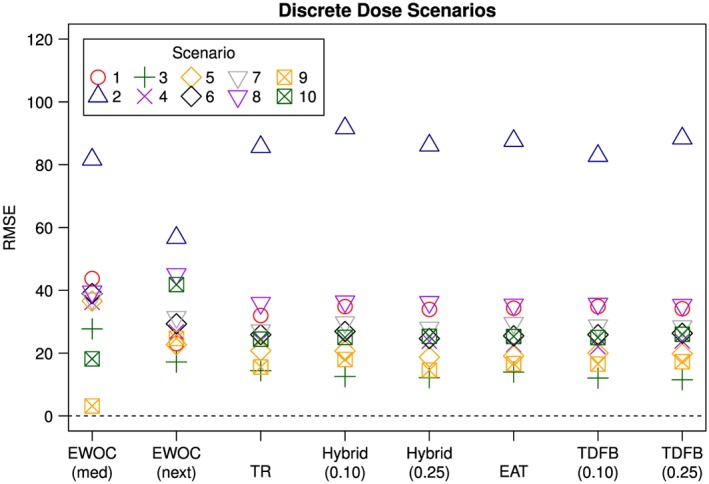
Root mean square error (RMSE) of maximum tolerated dose recommendations per approach for discrete dose scenarios. Black dashed line indicates an RMSE of 0. EAT, escalation in the absence of toxicity; EWOC, escalation with overdose control; TDFB, toxicity‐dependent feasibility bound; TR, Tighiouart and Rogatko design.

We simulated 1000 trials as per the description in subsection 3.3, but now, the dose in $L_{D}$ closest to $H_{n}^{-1}\left(\alpha_{n+1}\right)$ is used for experimentation and MTD recommendation when *n*=*N*. Figure 6 shows the EWOC approach with median MTD estimator to have poor accuracy in the majority of scenarios. Furthermore, the TR, Hybrid, EAT and TDFB approaches have accuracy scores of at least 0.90 in 5 out of 10 scenarios, whereas both EWOC approaches only have exceed this threshold in 3 out of 10 scenarios. When assessing the mean bias, Figure 7 shows the EWOC approaches are prone to positive and negative bias more so than the other approaches, as observed in the continuous dose scenarios. Figure 8 shows the approaches with escalating feasibility bounds have RMSEs clustered between 11.5 and 34.9, except for scenario 2. For the EWOC approach with median MTD estimator, the RMSE tends to cluster around 40, with no improvement in RMSE in scenario 2.

**Figure 9:**
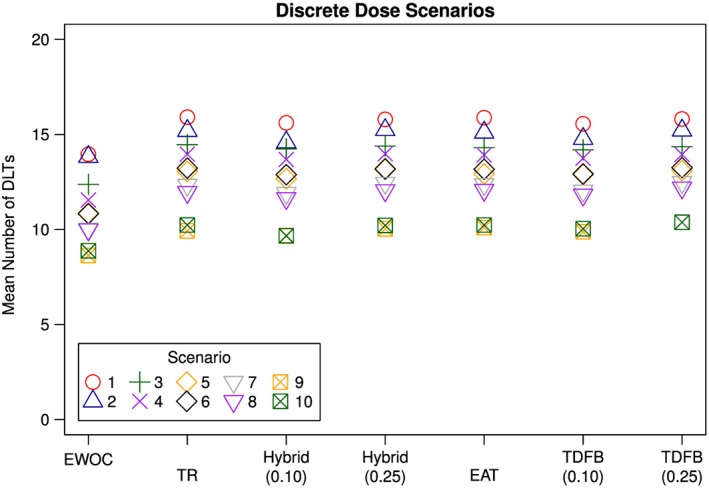
Mean number of dose‐limiting toxicity (DLTs) per approach for discrete dose scenarios. EAT, escalation in the absence of toxicity; EWOC, escalation with overdose control; TDFB, toxicity‐dependent feasibility bound; TR, Tighiouart and Rogatko design.

Figure 9 shows slightly fewer DLTs on average when using the EWOC approach than all other approaches, as seen under continuous dose scenarios. All other approaches offer similar mean numbers of DLTs. Whilst the EWOC approach doses fewer patients at overdoses, in scenarios 3–10, it also doses the lowest percentage of patients at the MTD (Table S3); for scenarios 1 and 2, where the MTD is the lowest dose level, the EWOC is the best performing approach (66.1*%* and 40.2*%* experimentation at the MTD, respectively). Otherwise, amongst the approaches that guarantee coherent dose escalation, the TDFB approach with $\alpha_{\min}=0.25$ and the EAT approach are the best performers (for scenarios 3–8 and 9–10, respectively). When including comparison to approaches that do not guarantee coherent escalation, the Hybrid design with $\alpha_{\min}=0.25$ provides slightly more experimentation at the MTD compared to the TDFB approach with the same $\alpha_{\min}$(0.2–1% across scenarios 3–10).

### 4.3 Sensitivity analyses

We also investigated the performance of each approach under model misspecification. Two true dose‐toxicity scenarios were generated from the power model $\pi \left(x^{*};\beta \right)={\left(x^{*}\right)}^{\exp(\beta )}$ and hyperbolic tangent model $\pi \left(x^{*};\beta \right)={\left(\frac{\tanh(x^{*})+1}{2}\right)}^{\exp(\beta )}$, assuming an MTD of 250mg/m^2^ (Figure S2 and Table S5); here $x^{*}\in \left[0,1\right]$ is the standardised dose mapped from $x\in \left[x_{\min},x_{\max}\right]$. We simulated 1000 trials as per the description in subsection 3.3. Results for these simulations are provided as supplementary material (Table S6 and Table S7 for continuous dose interval, Table S8 and Table S9 for discrete dose set). We found that for both scenarios the EWOC approach with a fixed feasibility bound dosed the second‐highest proportion of patients close to the true MTD (beaten by TR design under power model scenario and Hybrid ( $\alpha_{\min}=0.25$) under hyperbolic tangent model scenario), but dosed the lowest proportion of patients at overdoses. Coherence violations were observed under the TR and Hybrid designs. For MTD recommendations, the EAT approach (under power model) and TDFB ( $\alpha_{\min}=0.25$) provided most MTD recommendations in DLT probability interval [0.30,0.35), though the TDFB ( $\alpha_{\min}=0.25$) approach had the lowest mean bias in both scenarios. For discrete doses, all approaches exhibited similar experimentation at the MTD, with the EWOC providing the lowest proportion of patients experiencing overdoses. Coherence violations were only observed under the TR design. For recommendation, the EWOC approach performed the worst, giving the lowest proportion of recommendations at the true MTD and the largest bias, regardless of MTD estimator. All other approaches performed similarly.

## 5 Discussion

In this paper, we investigated the operating characteristics of EWOC approaches that used fixed and variable feasibility bounds to aid dose escalation and de‐escalation decisions. Although some approaches for increasing feasibility bounds mid‐trial have been used in practice, they have not been studied in detail in a comparative manner. Furthermore, increasing a feasibility bound regardless of DLT responses can lead to incoherent dose‐escalations; ideally, a dose‐escalation approach that guarantees coherence in dose escalation and de‐escalation, whilst offering favourable operating characteristics regarding experimentation and MTD recommendation, should be used. We proposed a TDFB approach to satisfy these requirements and compared its performance with several dose‐escalation approaches that either guarantee or do not guarantee coherent dose escalation.

In our simulations, incoherent dose escalation occurred rarely; in scenarios 1–10 where doses were selected from a continuous dose interval, the extent of incoherent escalation was not substantial (1−11mg/m^2^), yet in the discrete dose scenarios, an incoherent escalation was an increase in dose by 50mg/m^2^. Whilst the amounts witnessed here are not relatively large, Neuenschwander *et al.* 32 describe a trial using the CRM in which, after observing two DLTs in two patients at 25 mg, the model recommended dosing new patients at 40 mg, an increase of 60%; this was in part due to escalating from 10 to 25 mg and skipping two planned dose levels. Incoherent escalations may dissuade clinicians from using EWOC‐style approaches with increasing feasibility bounds and other model‐based designs in practice.

Coherent escalation behaviour is rarely emphasised in the literature on dose escalation designs 20, 22, 23, yet by its definition it is a serious ethical issue. An ideal phase I trial design should aim to dose as many patients at doses close to the TTL and recommend the correct MTD as often as possible for future clinical trials. However, a practically useful design should be able to incorporate sensible dose‐escalation behaviour as well, so clinicians can be confident in implementing it in practice repeatedly on a long‐term basis. The coherent approaches studied in this paper require minimal extra effort to implement in practice compared to approaches already used in trials that escalate the feasibility bound. Therefore, a design that guarantees coherent dose escalation with comparable or superior operating characteristics relative to existing approaches can and should be used.

The only approaches that guarantee coherence under the EWOC approach are the original EWOC approach with fixed *α*, the EAT approach and the TDFB approach. In our simulation studies, both the EAT and TDFB approaches showed comparable MTD recommendation percentages for doses around the TTL relative to other approaches with increasing feasibility bounds, as well as similar bias and RMSE; this was also the case for simulations conducted under two different forms of model misspecification. Both the EAT and TDFB approaches offer comparable performance to approaches that do not guarantee coherent dose escalation. It is interesting to note however that the EWOC approach seems to do moderately well with respect to MTD recommendation when the true MTD is close to the extreme ends of the dose range. However, the posterior median MTD estimator is positively biased, whereas the next dose estimator tends to be negatively biased. Therefore, using the posterior median MTD estimator under the original EWOC approach is not advised, given the poor MTD recommendation accuracy.

A review of 1235 phase I cancer trials published between 1991 and 2006 showed only three trials implemented an EWOC approach 7, whilst another review of phase I cancer trials published between 2007 and 2008 yielded no reported usage of the EWOC approach 1. However, Rogatko and Tighiouart 28 found 17 phase I trials that implemented EWOC approaches between 2001 and 2013, which shows that the use of more advanced designs for phase I trials is slowly increasing. Several papers have identified the primary reasons for the dearth of model‐based dose‐escalation methods in practice, and others have attempted to provide useful recommendations for conducting adaptive model‐based phase I trials 35, 36, 37. We hope the descriptive and investigative work in this paper is used to improve the design of clinical trials and to provide further guidance for using EWOC‐based approaches in future phase I cancer trials.

## Supporting information

Table S1: Experimentation percentages for all scenarios with continuous dose interval (TTL θ = 0:33). Denominators (number of times feasibility bound increased across 1000 trials) for approaches not guaranteeing coherence: TR design, 5000; Hybrid design, 19000.
Table S2: Recommendation percentages, bias and RMSE for all scenarios with continuous dose interval (TTL η = 0:33). For all approaches except EWOC, posterior median of MTD equals next dose to be given.
Table S3: Experimentation percentages for discrete dose scenarios (TTL θ= 0:33). Denominators (number of times feasibility bound increased across 1000 trials) for approaches not guaranteeing coherence: TR design, 5000; Hybrid design, 19000.
Table S4: Recommendation percentages, bias and RMSE for discrete dose scenarios (TTL θ = 0:33). For all approaches except EWOC, posterior median of MTD equals next dose to be given.
Table S5: Dose‐toxicity scenarios (for both continuous dose interval and discrete dose settings) for sensitivity analysis. MTDs are shown in bold.
Table S6: Experimentation percentages for scenarios specified under power and hyperbolic tangent models with continuous dose interval (TTL θ = 0:33). Denominators (number of times feasibility bound increased across 1000 trials) for approaches not guaranteeing coherence: TR design, 5000; Hybrid design, 19000.
Table S7: Recommendation percentages, bias and RMSE for scenarios specified under the power and hyperbolic tangent models with continuous dose interval (TTL θ = 0:33). For all approaches except EWOC, posterior median of MTD equals next dose to be given.
Table S8: Experimentation percentages for scenarios specified under power and hyperbolic tangent models with discrete doses (TTL θ = 0:33). Denominators (number of times feasibility bound increased across 1000 trials) for approaches not guaranteeing coherence: TR design, 5000; Hybrid design, 19000.
Table S9: Recommendation percentages, bias and RMSE for scenarios specified under power and hyperbolic tangent models with discrete doses (TTL θ = 0:33). For all approaches except EWOC, posterior median of MTD equals next dose to be given.
Figure S1: Prior dose‐toxicity relationships from uniform priors on γ and *ρ*_0_, and bivariate normal priors on the vector (*β*_0_(*β*_1_)). Lines in plots include mean (solid line), median (dotted) and 95% credibility interval (dashed).
Figure S2: Dose‐toxicity scenarios for continuous dose interval (left plot) and discrete doses (right plot) used in sensitivity analysis. TTL θ = 0:33 (blue dashed horizontal line).

## References

[1] Le Tourneau C , Lee JJ , Siu LL . Dose escalation methods in phase I cancer clinical trials. Journal of the National Cancer Institute 2009; 101(10):708–720. https://www.ncbi.nlm.nih.gov/pmc/articles/PMC2684552/?tool=pmcentrez. 19436029 10.1093/jnci/djp079 PMC2684552

[2] Babb JS , Rogatko A . Bayesian methods for phase I cancer clinical trials In Advances in clinical trial biostatistics, Geller NL (ed.).Marcel Dekker: New York, NY, 2004; 1–40.

[3] Green S , Benedetti J , Crowley J . Clinical Trials in Oncology 2nd edn. Chapman & Hall/CRC: New York, NY, 2003.

[4] Babb JS , Rogatko A , Zacks S . Cancer phase I clinical trials: efficient dose escalation with overdose control. Statistics in Medicine 1998; 17(10):1103–1120. 9618772 10.1002/(sici)1097-0258(19980530)17:10<1103::aid-sim793>3.0.co;2-9

[5] Carter SK . Study design principles for the clinical evaluation of new drugs as developed by the chemotherapy programme of the National Cancer Institute In The design of clinical trials in cancer therapy, Staquet MJ (ed.)Editions Scientifique Europe: Brussels, Belgium, 1973; 242–289.

[6] Storer BE . Design and analysis of phase I clinical trials. Biometrics 1989; 45(3):925–937. 2790129

[7] Rogatko A , Schoeneck D , Jonas W , Tighiouart M , Khuri FR , Porter A . Translation of innovative designs into phase I trials. Journal of Clinical Oncology 2007; 25(31):4982–4986. 17971597 10.1200/JCO.2007.12.1012

[8] Penel N , Isambert N , Leblond P , Ferte C , Duhamel A , Bonneterre J . “Classical 3 + 3 design" versus “accelerated titration designs": analysis of 270 phase 1 trials investigating anti‐cancer agents. Investigational New Drugs 2009; 27(6):552–556. 19132294 10.1007/s10637-008-9213-5

[9] Kang S‐H , Ahn CW . The expected toxicity rate at the maximum tolerated dose in the standard phase I cancer clinical trial design. Drug Information Journal 2001; 35(8):1189–1199.

[10] Kang S‐H , Ahn CW . An investigation of the traditional algorithm‐based designs for phase 1 cancer clinical trials. Drug Information Journal 2002; 36:865–873.

[11] He W , Liu J , Binkowitz B , Quan H . A model‐based approach in the estimation of the maximum tolerated dose in phase I cancer clinical trials. Statistics in Medicine 2006; 25(12):2027–2042. https: //www.ncbi.nlm.nih.gov/pubmed/16025542. 16025542 10.1002/sim.2334

[12] O'Quigley J , Zohar S . Experimental designs for phase I and phase I/II dose‐finding studies. British Journal of Cancer 2006; 94(5):609–613. https://www.pubmedcentral.nih.gov/articlerender.fcgi?artid=2374235&tool=pmcentrez&rendertype=abstract. 16434987 10.1038/sj.bjc.6602969 PMC2374235

[13] Chen Z , Krailo MD , Sun J , Azen SP . Range and trend of expected toxicity level (ETL) in standard A + B designs: a report from the Children's Oncology Group. Contemporary Clinical Trials 2009; 30(2):123–128. 19000782 10.1016/j.cct.2008.10.006

[14] Onar A , Kocak M , Boyett JM . Continual reassessment method vs. traditional empirically based design: modifications motivated by Phase I trials in pediatric oncology by the Pediatric Brain Tumor Consortium. Journal of Biopharmaceutical Statistics 2009; 19(3):437–455. https://www.ncbi.nlm.nih.gov/pmc/articles/PMC2976658/?tool= pmcentrez. 19384687 10.1080/10543400902800486 PMC2976658

[15] Onar‐Thomas A , Xiong Z . A simulation‐based comparison of the traditional method, Rolling‐6 design and a frequentist version of the continual reassessment method with special attention to trial duration in pediatric phase I oncology trials. Contemporary Clinical Trials 2010; 31(3):259–270. 20298812 10.1016/j.cct.2010.03.006 PMC2863035

[16] Babb JS , Rogatko A . Patient specific dosing in a cancer phase I clinical trial. Statistics in Medicine 2001; 20:2079–2090. 11439422 10.1002/sim.848

[17] Cheng JD , Babb JS , Langer C , Aamdal S , Robert F , Engelhardt LR , Fernberg O , Schiller J , Forsberg G , Alpaugh RK . Individualized patient dosing in phase I clinical trials: the role of escalation with overdose control in PNU214936. Journal of Clinical Oncology 2004; 22(4):602–609. 14966084 10.1200/JCO.2004.12.034

[18] Tighiouart M , Rogatko A . Dose finding with escalation with overdose control (EWOC) in cancer clinical trials. Statistical Science 2010; 25(2):217–226.

[19] Chu P‐L , Lin Y , Shih WJ . Unifying CRM and EWOC designs for phase I cancer clinical trials. Journal of Statistical Planning and Inference 2009; 139(3):1146–1163.

[20] Cheung YK . Coherence principles in dose‐finding studies. Biometrika 2005; 92(4):863–873.

[21] Cheung YK . Dose Finding by the Continual Reassessment Method. Chapman & Hall/CRC Biostatistics Series, Taylor and Francis: Boca Raton, FL, 2011.

[22] Bartroff J , Lai TL . Incorporating individual and collective ethics into phase I cancer trial designs. Biometrics 2011; 67(2):596–603. 20731643 10.1111/j.1541-0420.2010.01471.x PMC4485382

[23] Wheeler GM . Incoherent dose‐escalation in phase I trials using the escalation with overdose control approach. Statistical Papers 2016:1–11. [Epub ahead of print] DOI:10.1007/s00362‐016‐0790‐7. 10.1007/s00362-016-0790-7 PMC5985932 29875549

[24] Kadane JB , Dickey JM , Winkler RL , Smith WS , Peters C , Dickey M , Winkler L , Peters SC . Interactive elicitation of opinion for a normal linear model. Journal of the American Statistical Association 1980; 75(372):845–854.

[25] Tighiouart M , Rogatko A , Babb JS . Flexible Bayesian methods for cancer phase I clinical trials. Dose escalation with overdose control. Statistics in Medicine 2005; 24(14):2183–2196. 15909291 10.1002/sim.2106

[26] Berry SM , Carlin BP , Lee JJ , Mueller P . Bayesian Adaptive Methods for Clinical Trials. Chapman & Hall/CRC Biostatistics Series, Taylor and Francis: Boca Raton, FL, 2010.

[27] Tighiouart M , Liu Y , Rogatko A . Escalation with overdose control using time to toxicity for cancer phase I clinical trials. PloS ONE 2014; 9(3):e93070, 1–13. 24663812 10.1371/journal.pone.0093070 PMC3963973

[28] Rogatko A , Tighiouart M . Designing a dose finding trial using EWOC, 2013 https://biostatistics.csmc.edu/ewoc/download/Designing{\_}a{\_}Dose{\_}Finding{\_}Trial{\_}using{\_}EWOC{\&}Appendix.pdf.

[29] O'Quigley J , Pepe M , Fisher L . Continual reassessment method: a practical design for phase 1 clinical trials in cancer. Biometrics 1990; 46(1):33–48. 2350571

[30] O'Quigley J , Shen LZ . Continual reassessment method: a likelihood approach. Biometrics 1996; 52(2):673–684. 8672707

[31] Jia X , Lee SM , Cheung YK . Characterization of the likelihood continual reassessment method. Biometrika 2014; 101(3):599–612. https://biomet.oxfordjournals.org/cgi/doi/10.1093/biomet/asu012.

[32] Neuenschwander B , Branson M , Gsponer T . Critical aspects of the Bayesian approach to phase I cancer trials. Statistics in Medicine 2008; 27(13):2420–2439. 18344187 10.1002/sim.3230

[33] Sweeting M , Mander A , Sabin T . bcrm: Bayesian continual reassessment method designs for phase I dose‐finding trials. Journal of Statistical Software 2013; 54(13):1–26.

[34] Plummer M . rjags: Bayesian graphical models using MCMC, 2016 Available from: https://cran.r‐project.org/ package=rjags, Accessed 01/19/2017.

[35] Paoletti X , Baron B , Schöffski P , Fumoleau P , Lacombe D , Marreaud S , Sylvester R . Using the continual reassessment method: lessons learned from an EORTC phase I dose finding study. European Journal of Cancer 2006; 42(10):1362–8. https://www.ncbi.nlm.nih.gov/pubmed/16740385. 16740385 10.1016/j.ejca.2006.01.051

[36] Bailey SM , Neuenschwander B , Laird G , Branson M . A Bayesian case study in oncology phase I combination dose‐finding using logistic regression with covariates. Journal of Biopharmaceutical Statistics 2009; 19(3):469–484. https://www.ncbi.nlm.nih.gov/pubmed/19384689. 19384689 10.1080/10543400902802409

[37] Harrington JA , Wheeler GM , Sweeting MJ , Mander AP , Jodrell DI . Adaptive designs for dual‐agent phase I dose‐escalation studies. Nature Reviews Clinical Oncology 2013; 10(5):277–88. 10.1038/nrclinonc.2013.35 23507740

