# Supplementary material for: Toxicity‐dependent feasibility bounds for the escalation with overdose control approach in phase I cancer trials
Source: Stat Med. 2017 Mar 15;36(16):2499–513. doi: 10.1002/sim.7280 (PMC5462100; doi:10.1002/sim.7280)
Supplement: Supplementary file 1 — Table S1: Experimentation percentages for all scenarios with continuous dose interval (TTL θ = 0:33). Denominators (number of times feasibility bound increased across 1000 trials) for approaches not guaranteeing coherence: TR design, 5000; Hybrid design, 19000. Table S2: Recommendation percentages, bias and RMSE for all scenarios with continuous dose interval (TTL η = 0:33). For all approaches except EWOC, posterior median of MTD equals next dose to be given. Table S3: Experimentation percentages for discrete dose scenarios (TTL θ= 0:33). Denominators (number of times feasibility bound increased across 1000 trials) for approaches not guaranteeing coherence: TR design, 5000; Hybrid design, 19000. Table S4: Recommendation percentages, bias and RMSE for discrete dose scenarios (TTL θ = 0:33). For all approaches except EWOC, posterior median of MTD equals next dose to be given. Table S5: Dose‐toxicity scenarios (for both continuous dose interval and discrete dose settings) for sensitivity analysis. MTDs are shown in bold. Table S6: Experimentation percentages for scenarios specified under power and hyperbolic tangent models with continuous dose interval (TTL θ = 0:33). Denominators (number of times feasibility bound increased across 1000 trials) for approaches not guaranteeing coherence: TR design, 5000; Hybrid design, 19000. Table S7: Recommendation percentages, bias and RMSE for scenarios specified under the power and hyperbolic tangent models with continuous dose interval (TTL θ = 0:33). For all approaches except EWOC, posterior median of MTD equals next dose to be given. Table S8: Experimentation percentages for scenarios specified under power and hyperbolic tangent models with discrete doses (TTL θ = 0:33). Denominators (number of times feasibility bound increased across 1000 trials) for approaches not guaranteeing coherence: TR design, 5000; Hybrid design, 19000. Table S9: Recommendation percentages, bias and RMSE for scenarios specified under power and hyperbolic [file SIM-36-2499-s001.zip › TDFB MANUSCRIPT SUPPLEMENTARY MATERIAL ONLY - CLEAN - 19012017.pdf]

Supplementary Material

Specifying prior distributions

We use the `fitNorm2` function in the R package `prada` [1] to map the uniform priors on γ and ρ_0 to a bivariate Normal distribution on the vector $(\beta_0, \log(\beta_1))^T$. The R code for this procedure is below.

```
install.packages("mvtnorm")
source("https://bioconductor.org/biocLite.R")
biocLite("prada")
library(mvtnorm)
library(prada)

### Functions
# ptox.rhogam.fn - probability of toxicity for dose range based on gamma and rho_0 parameters
ptox.rhogam.fn<-function(dose, gamp, rhop, xmin, xmax, theta, N){
  ptox<-matrix(NA, nrow=N, ncol=length(dose))
  for(i in 1:length(dose)){
    lin.pred<-(gam.prior*qlogis(rho.prior) - xmin*qlogis(theta) + (qlogis(theta) -
    qlogis(rho.prior))*dose[i])/(gam.prior-xmin)
    ptox[,i]<-plogis(lin.pred)
  }
  ptox
}

# ptox.intslo.fn - probability of toxicity for dose range based on intercept and slope parameters
ptox.intslo.fn<-function(dose, int, slo, xmin, xmax, N){
  ptox<-matrix(NA, nrow=N, ncol=length(dose))
  for(i in 1:length(dose)){
    lin.pred<-int+slo*(dose[i])
    ptox[,i]<-plogis(lin.pred)
  }
  ptox
}

# plot.fn - plot function to display mean dose-toxicity and various quantiles
plot.fn<-function(ptox, dose, title){
  plot(dose, apply(ptox, 2, mean), ylim=c(0,1), las=1, xlab=expression("Dose (mg/"*m^2*")"),
  ylab="Probability of DLT", main=title, type="l", lwd=2, lty=1)
  lines(dose, apply(ptox, 2, quantile, probs=c(0.025, 0.975)), lty=2)
  lines(dose, apply(ptox, 2, quantile, probs=0.5), lty=3)
}

### Code to be run
N<-10000
xmin<-140
xmax<-425
dose<-seq(xmin, xmax, by=1)
theta<-1/3
gam.prior<-runif(N, xmin, xmax)
rho.prior<-runif(N, 0, theta)

# Parameterise intercept and slope in terms of gamma and rho
int.x<-(gam.prior*qlogis(rho.prior) - xmin*qlogis(theta))/(gam.prior-xmin)
slo.x<-(qlogis(theta) - qlogis(rho.prior))/(gam.prior-xmin)

# fit bivariate normal distribution to reparameterised intercept and slope distributions
foo<-fitNorm2(int.x[1:N], log(slo.x[1:N]), method="cov.rob")
```

```
mu<-foo$mu
Sigma<-foo$S
intslo<-rmvnorm(N, mu, Sigma)
intercept<-intslo[,1]
slope<-exp(intslo[,2])

# dose-toxicity relationship for 1) Uniform priors and 2) Bivariate Normal prior
ptox1<-ptox.rhogam.fn(dose, gam.prior, rho.prior, xmin, xmax, theta, N)
ptox2<-ptox.intslo.fn(dose, intercept, slope, xmin, xmax, N)

# Plot to show priors side-by-side
par(mfrow=c(1, 2))
plot.fn(ptox1, dose=dose, title=expression("Priors on"~gamma~"and"~rho[0]))
plot.fn(ptox2, dose=dose, title=expression("Priors on"~beta[0]~"and"~beta[1]))
```

The resultant plot comparing prior beliefs for the dose-toxicity relationship under uniform priors and a bivariate Normal prior on $(\beta_0, \log(\beta_1))^T$ is shown in Figure S1.

References

[1] Hahne F, Huber W, Ruschhaupt M, Toedling J, Barry J. prada: Data analysis for cell-based functional assays 2016. URL https://bioconductor.org/packages/release/bioc/html/prada.html.

Scenario/ Model	Probability of DLT							Mean No. DLTs	Coherence Violations
	$[0, 0.15]$	$(0.15, 0.25]$	$(0.25, 0.30]$	$(0.30, 0.35]$	$(0.35, 0.40]$	$(0.40, 0.50]$	$(0.50, 1]$		
**Scenario 1** ($\gamma = 165, \rho_0 = 0.25$)									
EWOC	0.0	25.7	16.1	18.8	12.8	15.6	11.0	13.8	0
TR	0.0	12.2	11.3	17.7	16.0	24.2	18.6	15.8	12
Hybrid ($\alpha_{\min} = 0.10$)	0.0	14.8	10.1	14.2	19.4	24.8	16.6	15.5	11
Hybrid ($\alpha_{\min} = 0.25$)	0.0	10.4	11.6	17.0	17.4	23.7	19.8	15.9	14
EAT	0.0	13.2	10.5	14.9	16.4	25.6	19.4	15.9	0
TDFB ($\alpha_{\min} = 0.10$)	0.0	16.8	9.2	14.2	16.0	25.9	18.0	15.5	0
TDFB ($\alpha_{\min} = 0.25$)	0.0	11.8	10.3	16.5	16.2	23.6	21.6	15.7	0
**Scenario 2** ($\gamma = 175, \rho_0 = 0.30$)									
EWOC	0.0	0.0	22.6	30.2	28.9	17.4	0.9	13.9	0
TR	0.0	0.0	10.1	22.3	31.6	31.0	5.0	15.0	16
Hybrid ($\alpha_{\min} = 0.10$)	0.0	0.0	18.3	26.7	26.6	24.9	3.5	14.7	6
Hybrid ($\alpha_{\min} = 0.25$)	0.0	0.0	9.4	21.7	30.8	33.4	4.7	15.2	12
EAT	0.0	0.0	15.7	20.8	28.0	29.3	6.2	15.0	0
TDFB ($\alpha_{\min} = 0.10$)	0.0	0.0	19.2	24.5	26.3	25.3	4.8	14.6	0
TDFB ($\alpha_{\min} = 0.25$)	0.0	0.0	9.6	20.2	30.2	34.7	5.3	15.2	0
**Scenario 3** ($\gamma = 200, \rho_0 = 0.03$)									
EWOC	17.6	25.0	11.5	12.2	8.2	11.9	13.5	12.2	0
TR	11.5	18.5	11.0	12.6	10.2	14.7	21.5	14.5	13
Hybrid ($\alpha_{\min} = 0.10$)	11.1	19.7	10.6	10.9	12.9	12.6	22.2	14.4	4
Hybrid ($\alpha_{\min} = 0.25$)	10.9	18.3	10.7	11.7	11.7	16.4	20.4	14.6	7
EAT	11.9	18.6	14.1	11.7	9.7	11.7	22.3	14.4	0
TDFB ($\alpha_{\min} = 0.10$)	11.9	19.8	10.8	11.2	10.1	13.8	22.5	14.4	0
TDFB ($\alpha_{\min} = 0.25$)	12.4	19.7	10.5	12.1	10.2	12.8	22.2	14.5	0
**Scenario 4** ($\gamma = 250, \rho_0 = 0.05$)									
EWOC	9.8	29.4	16.6	14.9	11.0	10.2	8.0	11.8	0
TR	6.7	16.7	14.0	15.9	14.5	15.9	16.1	14.2	17
Hybrid ($\alpha_{\min} = 0.10$)	11.5	14.3	13.3	13.8	14.1	17.4	15.6	13.7	8
Hybrid ($\alpha_{\min} = 0.25$)	5.4	17.6	13.5	15.0	15.5	18.8	14.4	14.2	6
EAT	10.9	12.6	14.3	13.5	13.5	18.5	16.6	14.0	0
TDFB ($\alpha_{\min} = 0.10$)	11.7	15.4	12.8	13.8	13.8	15.9	16.6	13.8	0
TDFB ($\alpha_{\min} = 0.25$)	5.9	18.5	13.9	14.9	14.8	16.5	15.5	14.1	0
**Scenario 5** ($\gamma = 300, \rho_0 = 0.001$)									
EWOC	25.3	25.4	12.8	8.4	7.8	9.4	10.8	11.0	0
TR	17.8	20.4	12.5	8.7	8.7	12.0	20.0	13.2	6
Hybrid ($\alpha_{\min} = 0.10$)	22.1	16.2	11.0	10.0	8.4	12.2	20.2	12.8	3
Hybrid ($\alpha_{\min} = 0.25$)	16.4	21.4	11.4	8.2	11.2	12.5	18.8	13.3	6
EAT	18.9	21.0	11.6	8.0	10.2	11.0	19.2	12.9	0
TDFB ($\alpha_{\min} = 0.10$)	20.6	18.7	10.5	8.6	10.3	12.2	19.0	12.9	0
TDFB ($\alpha_{\min} = 0.25$)	18.6	19.5	11.7	8.4	9.0	12.8	20.0	13.3	0
**Scenario 6** ($\gamma = 300, \rho_0 = 0.02$)									
EWOC	15.6	30.4	16.8	12.9	9.2	9.9	5.1	10.9	0
TR	10.7	18.5	14.6	14.1	11.5	16.4	14.1	13.3	21
Hybrid ($\alpha_{\min} = 0.10$)	16.5	15.9	12.8	13.3	11.4	16.4	13.6	12.9	6
Hybrid ($\alpha_{\min} = 0.25$)	9.4	20.3	13.1	13.1	14.1	15.8	14.1	13.3	13
EAT	14.0	15.9	14.3	12.9	12.3	16.2	14.4	13.1	0
TDFB ($\alpha_{\min} = 0.10$)	14.6	16.3	13.8	12.0	11.4	17.0	15.0	13.0	0
TDFB ($\alpha_{\min} = 0.25$)	9.5	17.8	15.6	13.2	12.0	17.4	14.5	13.3	0
**Scenario 7** ($\gamma = 350, \rho_0 = 0.01$)									
EWOC	22.8	27.7	14.9	12.1	8.3	9.7	4.4	10.1	0
TR	17.9	18.1	13.3	11.9	10.9	15.2	12.7	12.5	12
Hybrid ($\alpha_{\min} = 0.10$)	20.2	18.6	10.5	12.9	10.9	14.5	12.4	11.9	3
Hybrid ($\alpha_{\min} = 0.25$)	15.4	19.4	12.2	13.8	11.1	16.0	12.2	12.5	7
EAT	16.6	19.4	13.0	11.9	11.6	14.0	13.5	12.4	0
TDFB ($\alpha_{\min} = 0.10$)	20.0	18.1	11.0	12.7	10.6	14.8	12.8	12.1	0
TDFB ($\alpha_{\min} = 0.25$)	16.1	19.8	12.0	12.9	11.6	14.5	13.1	12.6	0
**Scenario 8** ($\gamma = 350, \rho_0 = 0.05$)									
EWOC	16.7	35.4	17.9	13.5	8.3	6.8	1.5	10.1	0
TR	12.8	19.9	14.9	15.3	13.1	15.2	8.8	12.2	12
Hybrid ($\alpha_{\min} = 0.10$)	17.0	18.5	13.6	15.3	12.6	14.6	8.4	11.8	5
Hybrid ($\alpha_{\min} = 0.25$)	9.2	21.8	15.4	16.9	13.5	15.3	8.0	12.3	5
EAT	13.3	16.6	15.7	15.4	13.6	15.6	9.9	12.4	0
TDFB ($\alpha_{\min} = 0.10$)	15.3	19.8	13.4	15.4	12.5	14.6	9.0	12.0	0
TDFB ($\alpha_{\min} = 0.25$)	9.6	20.3	16.2	16.6	14.0	15.4	7.9	12.4	0
**Scenario 9** ($\gamma = 400, \rho_0 = 0.001$)									
EWOC	32.5	25.6	9.3	9.4	7.0	16.0	0.0	9.3	0
TR	23.8	18.3	7.9	8.4	8.8	32.9	0.0	11.5	6
Hybrid ($\alpha_{\min} = 0.10$)	28.0	16.2	8.2	7.9	8.5	31.3	0.0	10.9	2
Hybrid ($\alpha_{\min} = 0.25$)	22.4	17.3	10.4	8.8	8.2	32.9	0.0	11.6	6
EAT	25.1	16.4	9.3	11.2	6.9	31.1	0.0	11.4	0
TDFB ($\alpha_{\min} = 0.10$)	25.7	16.6	9.0	8.7	6.9	33.1	0.0	11.2	0
TDFB ($\alpha_{\min} = 0.25$)	20.5	20.6	9.6	9.3	7.2	32.9	0.0	11.6	0
**Scenario 10** ($\gamma = 400, \rho_0 = 0.03$)									
EWOC	24.7	34.7	14.3	11.2	15.2	0.0	0.0	9.1	0
TR	19.0	20.6	12.5	13.3	34.6	0.0	0.0	11.0	5
Hybrid ($\alpha_{\min} = 0.10$)	22.7	19.0	12.6	11.6	34.1	0.0	0.0	10.4	6
Hybrid ($\alpha_{\min} = 0.25$)	16.0	20.8	14.5	13.7	35.0	0.0	0.0	11.0	4
EAT	16.6	19.5	13.6	14.1	36.1	0.0	0.0	11.1	0
TDFB ($\alpha_{\min} = 0.10$)	20.4	18.8	12.7	11.4	36.7	0.0	0.0	10.6	0
TDFB ($\alpha_{\min} = 0.25$)	16.1	22.3	14.1	12.3	35.1	0.0	0.0	11.2	0

Table S1: Experimentation percentages for all scenarios with continuous dose interval (TTL $\theta = 0.33$). Denominators (number of times feasibility bound increased across 1000 trials) for approaches not guaranteeing coherence: TR design, 5000; Hybrid design, 19000.

Scenario/ Model	Probability of DLT							Mean Bias	RMSE
	$[0, 0.15]$	$(0.15, 0.25]$	$(0.25, 0.30]$	$(0.30, 0.35]$	$(0.35, 0.40]$	$(0.40, 0.50]$	$(0.50, 1]$		
**Scenario 1** ($\gamma = 165, \rho_0 = 0.25$)									
EWOC (median)	0.0	5.9	10.4	17.4	22.7	31.0	12.6	14.8	28.2
EWOC (next dose)	0.0	23.9	22.5	24.7	16.6	11.7	0.6	-5.1	18.7
TR	0.0	6.7	14.7	24.5	25.7	24.9	3.5	6.8	20.2
Hybrid ($\alpha_{\min} = 0.10$)	0.0	6.1	10.9	24.6	27.4	25.5	5.5	9.3	22.6
Hybrid ($\alpha_{\min} = 0.25$)	0.0	5.3	16.1	25.7	23.8	25.0	4.1	7.1	20.7
EAT	0.0	5.6	11.9	26.8	26.1	24.0	5.6	8.5	21.8
TDFB ($\alpha_{\min} = 0.10$)	0.0	5.5	11.6	22.4	26.1	29.3	5.1	9.9	22.5
TDFB ($\alpha_{\min} = 0.25$)	0.0	6.8	10.8	24.0	26.3	25.8	6.3	9.4	22.6
**Scenario 2** ($\gamma = 175, \rho_0 = 0.30$)									
EWOC (median)	0.0	0.0	11.1	32.2	34.4	21.3	1.0	30.0	56.7
EWOC (next dose)	0.0	0.0	26.2	38.1	26.1	9.4	0.2	6.7	40.8
TR	0.0	0.0	6.3	24.6	36.7	29.3	3.1	44.4	67.9
Hybrid ($\alpha_{\min} = 0.10$)	0.0	0.0	7.9	27.4	36.4	26.8	1.5	38.6	63.2
Hybrid ($\alpha_{\min} = 0.25$)	0.0	0.0	6.1	26.6	35.2	29.7	2.4	43.7	67.0
EAT	0.0	0.0	6.5	25.7	34.5	30.7	2.6	45.9	70.3
TDFB ($\alpha_{\min} = 0.10$)	0.0	0.0	8.1	26.1	34.1	28.8	2.9	42.6	68.7
TDFB ($\alpha_{\min} = 0.25$)	0.0	0.0	6.4	24.8	33.3	33.1	2.4	46.5	69.3
**Scenario 3** ($\gamma = 200, \rho_0 = 0.03$)									
EWOC (median)	0.0	2.5	2.9	5.0	9.2	19.2	61.2	-20.0	25.4
EWOC (next dose)	5.2	30.8	17.6	16.4	12.1	12.0	5.9	-4.2	12.4
TR	1.8	22.5	17.4	18.0	16.6	16.0	7.7	-1.0	10.8
Hybrid ($\alpha_{\min} = 0.10$)	1.9	21.3	16.3	17.3	17.2	16.0	10.0	-0.1	11.5
Hybrid ($\alpha_{\min} = 0.25$)	1.1	22.4	18.1	19.3	17.0	14.9	7.2	-0.8	10.8
EAT	1.8	23.5	17.3	19.4	15.4	14.4	8.2	-1.3	10.7
TDFB ($\alpha_{\min} = 0.10$)	1.4	21.5	16.1	19.3	17.0	14.5	10.2	-0.2	11.2
TDFB ($\alpha_{\min} = 0.25$)	2.2	25.0	16.6	19.4	15.0	14.7	7.1	-0.6	10.9
**Scenario 4** ($\gamma = 250, \rho_0 = 0.05$)									
EWOC (median)	0.2	3.7	6.8	11.4	18.3	27.9	31.7	22.6	33.9
EWOC (next dose)	2.0	27.0	24.3	19.0	14.8	10.1	2.8	-8.6	22.8
TR	0.1	15.0	21.6	19.9	20.8	17.6	5.0	0.3	19.3
Hybrid ($\alpha_{\min} = 0.10$)	0.3	10.0	20.2	23.9	18.1	20.2	7.3	3.2	20.2
Hybrid ($\alpha_{\min} = 0.25$)	0.2	15.2	20.2	20.3	20.3	18.0	5.8	0.9	20.1
EAT	0.3	13.3	19.7	24.1	18.8	17.3	6.5	1.3	19.9
TDFB ($\alpha_{\min} = 0.10$)	0.1	11.8	20.8	20.9	21.6	18.0	6.8	2.5	20.2
TDFB ($\alpha_{\min} = 0.25$)	0.5	12.5	21.7	22.0	18.6	19.4	5.3	1.4	19.9
**Scenario 5** ($\gamma = 300, \rho_0 = 0.001$)									
EWOC (median)	0.1	0.8	2.4	2.3	5.5	15.7	73.2	30.6	36.4
EWOC (next dose)	8.9	28.7	18.4	12.4	13.0	12.1	6.5	-5.7	16.6
TR	4.6	23.8	19.5	13.3	14.5	14.7	9.6	-1.9	15.2
Hybrid ($\alpha_{\min} = 0.10$)	5.7	25.0	16.8	13.8	12.1	15.7	10.9	-2.1	15.8
Hybrid ($\alpha_{\min} = 0.25$)	4.2	24.4	17.8	14.1	12.4	17.3	9.8	-1.8	14.9
EAT	3.9	25.2	17.8	12.4	14.3	15.9	10.5	-1.5	15.1
TDFB ($\alpha_{\min} = 0.10$)	4.3	24.3	17.1	14.5	13.6	16.5	9.7	-1.7	14.9
TDFB ($\alpha_{\min} = 0.25$)	4.4	24.3	16.9	12.8	14.7	16.1	10.8	-1.6	15.2
**Scenario 6** ($\gamma = 300, \rho_0 = 0.02$)									
EWOC (median)	0.0	2.1	4.9	10.3	14.0	28.9	39.8	29.0	39.7
EWOC (next dose)	1.7	29.5	21.7	17.6	15.1	11.0	3.4	-8.5	23.6
TR	0.5	16.7	20.7	23.2	16.5	17.9	4.5	-0.7	21.0
Hybrid ($\alpha_{\min} = 0.10$)	0.8	15.6	20.0	19.7	18.5	19.3	6.1	0.4	22.1
Hybrid ($\alpha_{\min} = 0.25$)	0.3	16.2	20.4	19.1	18.3	18.4	7.3	1.0	22.3
EAT	0.5	17.3	19.5	19.7	18.3	19.1	5.6	-0.4	21.8
TDFB ($\alpha_{\min} = 0.10$)	0.5	14.2	21.7	21.2	18.6	18.8	5.0	0.3	20.6
TDFB ($\alpha_{\min} = 0.25$)	0.3	15.6	19.2	21.3	18.1	18.3	7.2	1.5	22.1
**Scenario 7** ($\gamma = 350, \rho_0 = 0.01$)									
EWOC (median)	0.0	3.0	5.1	7.9	13.0	30.2	40.8	31.3	41.7
EWOC (next dose)	3.2	29.3	21.1	17.1	14.8	11.6	2.9	-10.0	26.9
TR	0.7	20.1	19.4	18.3	17.0	17.4	7.1	-1.0	25.0
Hybrid ($\alpha_{\min} = 0.10$)	0.5	15.1	21.0	19.9	18.1	19.4	6.0	0.6	22.7
Hybrid ($\alpha_{\min} = 0.25$)	0.6	18.3	19.1	17.5	18.2	19.5	6.8	0.1	24.6
EAT	0.7	20.9	18.6	19.8	17.4	16.1	6.5	-1.9	24.3
TDFB ($\alpha_{\min} = 0.10$)	0.4	18.2	20.2	19.5	16.5	18.6	6.6	-0.3	24.4
TDFB ($\alpha_{\min} = 0.25$)	0.3	17.5	21.3	20.9	16.5	18.2	5.3	-1.0	23.7
**Scenario 8** ($\gamma = 350, \rho_0 = 0.05$)									
EWOC (median)	0.1	6.6	11.2	16.8	19.4	29.3	16.6	21.0	42.6
EWOC (next dose)	1.0	30.6	26.2	19.7	13.6	7.7	1.2	-20.2	40.5
TR	0.2	9.9	19.7	24.0	21.8	18.1	6.3	3.9	34.7
Hybrid ($\alpha_{\min} = 0.10$)	0.1	11.4	19.3	23.3	20.8	17.8	7.3	4.2	35.8
Hybrid ($\alpha_{\min} = 0.25$)	0.1	11.0	22.0	22.9	20.3	18.3	5.4	2.7	34.4
EAT	0.3	12.5	19.8	25.8	20.1	16.8	4.7	1.0	34.2
TDFB ($\alpha_{\min} = 0.10$)	0.0	11.9	20.0	25.2	20.5	17.0	5.4	2.1	34.3
TDFB ($\alpha_{\min} = 0.25$)	0.1	12.6	18.2	27.0	20.4	16.1	5.6	1.5	34.8
**Scenario 9** ($\gamma = 400, \rho_0 = 0.001$)									
EWOC (median)	0.0	1.8	2.8	5.5	6.5	83.4	0.0	19.9	22.5
EWOC (next dose)	6.3	30.8	17.5	12.8	10.9	21.7	0.0	-8.4	23.8
TR	2.3	21.5	14.6	15.9	12.9	32.8	0.0	-0.5	20.8
Hybrid ($\alpha_{\min} = 0.10$)	3.4	20.5	13.6	15.2	13.0	34.3	0.0	-0.4	21.0
Hybrid ($\alpha_{\min} = 0.25$)	2.5	20.6	16.7	15.3	12.8	32.1	0.0	-0.7	20.6
EAT	2.3	22.7	14.0	18.9	13.1	29.0	0.0	-1.7	20.6
TDFB ($\alpha_{\min} = 0.10$)	2.4	22.1	16.1	16.8	11.1	31.5	0.0	-1.5	20.5
TDFB ($\alpha_{\min} = 0.25$)	2.1	21.6	16.0	13.8	12.9	33.6	0.0	-0.3	20.4
**Scenario 10** ($\gamma = 400, \rho_0 = 0.03$)									
EWOC (median)	0.2	5.8	9.3	15.6	69.1	0.0	0.0	9.5	25.0
EWOC (next dose)	1.1	30.9	25.4	17.8	24.8	0.0	0.0	-21.9	40.3
TR	0.2	15.0	17.7	20.7	46.4	0.0	0.0	-3.2	28.8
Hybrid ($\alpha_{\min} = 0.10$)	0.1	12.7	16.6	21.7	48.9	0.0	0.0	-1.7	28.3
Hybrid ($\alpha_{\min} = 0.25$)	0.3	11.0	16.7	22.1	49.9	0.0	0.0	-0.6	27.5
EAT	0.3	14.7	19.4	20.3	45.3	0.0	0.0	-4.1	29.1
TDFB ($\alpha_{\min} = 0.10$)	0.3	14.2	16.8	20.6	48.1	0.0	0.0	-2.3	29.5
TDFB ($\alpha_{\min} = 0.25$)	0.2	17.4	18.2	18.2	46.0	0.0	0.0	-5.0	30.2

Table S2: Recommendation percentages, bias and RMSE for all scenarios with continuous dose interval (TTL $\theta = 0.33$). For all approaches except EWOC, posterior median of MTD equals next dose to be given.

Scenario/ Method	Dose Level d_1	d_2	d_3	d_4	d_5	d_6	Mean No. DLTs	Coherence Violations
**Scenario 1** ($\gamma = 165, \rho_0 = 0.25$)								
Probability of DLT	***0.28***	*0.47*	*0.66*	*0.82*	*0.91*	*0.96*		
EWOC	66.1	29.0	4.3	0.6	0.0	0.0	14.0	0
TR	46.7	44.3	8.2	0.7	0.0	0.0	15.9	0
Hybrid ($\alpha_{\min} = 0.10$)	46.8	44.6	8.0	0.6	0.0	0.0	15.6	1
Hybrid ($\alpha_{\min} = 0.25$)	46.0	45.0	7.8	1.1	0.0	0.0	15.8	0
EAT	47.2	42.7	8.5	1.5	0.1	0.0	15.9	0
TDFB ($\alpha_{\min} = 0.10$)	49.5	41.6	8.0	0.8	0.1	0.0	15.6	0
TDFB ($\alpha_{\min} = 0.25$)	47.1	43.0	8.5	1.3	0.1	0.0	15.8	0
**Scenario 2** ($\gamma = 175, \rho_0 = 0.30$)								
Probability of DLT	***0.31***	*0.36*	*0.41*	*0.46*	*0.52*	*0.57*		
EWOC	40.2	32.1	20.3	6.0	1.2	0.1	13.8	0
TR	24.3	30.6	25.7	13.8	4.3	1.4	15.2	0
Hybrid ($\alpha_{\min} = 0.10$)	28.7	29.2	23.9	12.3	4.6	1.4	14.6	0
Hybrid ($\alpha_{\min} = 0.25$)	22.3	29.4	28.4	14.2	4.6	1.2	15.2	1
EAT	26.7	28.2	24.3	13.9	5.0	2.0	15.1	0
TDFB ($\alpha_{\min} = 0.10$)	35.1	27.9	21.8	10.2	3.7	1.3	14.8	0
TDFB ($\alpha_{\min} = 0.25$)	22.2	29.9	26.1	14.7	5.3	1.7	15.2	0
**Scenario 3** ($\gamma = 200, \rho_0 = 0.03$)								
Probability of DLT	*0.05*	***0.33***	*0.84*	*0.98*	*1.00*	*1.00*		
EWOC	21.0	71.7	7.2	0.1	0.0	0.0	12.4	0
TR	12.4	75.5	11.9	0.2	0.0	0.0	14.5	0
Hybrid ($\alpha_{\min} = 0.10$)	13.2	74.7	11.8	0.3	0.0	0.0	14.2	0
Hybrid ($\alpha_{\min} = 0.25$)	11.1	76.7	11.8	0.4	0.0	0.0	14.4	0
EAT	13.2	74.0	12.1	0.6	0.0	0.0	14.3	0
TDFB ($\alpha_{\min} = 0.10$)	13.6	74.0	12.0	0.3	0.0	0.0	14.2	0
TDFB ($\alpha_{\min} = 0.25$)	12.1	76.0	11.6	0.4	0.0	0.0	14.4	0
**Scenario 4** ($\gamma = 250, \rho_0 = 0.05$)								
Probability of DLT	*0.06*	*0.15*	***0.33***	*0.58*	*0.79*	*0.92*		
EWOC	4.4	31.7	53.1	10.5	0.3	0.0	11.6	0
TR	3.9	17.1	56.9	20.1	1.9	0.1	14.0	0
Hybrid ($\alpha_{\min} = 0.10$)	6.8	14.9	56.0	20.2	2.1	0.1	13.7	0
Hybrid ($\alpha_{\min} = 0.25$)	3.3	16.4	58.2	20.4	1.7	0.0	14.0	0
EAT	6.2	15.6	56.6	19.4	2.1	0.1	13.9	0
TDFB ($\alpha_{\min} = 0.10$)	6.7	15.9	56.3	19.2	1.9	0.1	13.8	0
TDFB ($\alpha_{\min} = 0.25$)	3.8	17.6	57.7	18.7	2.2	0.1	14.0	0
**Scenario 5** ($\gamma = 300, \rho_0 = 0.001$)								
Probability of DLT	*0.00*	*0.01*	*0.07*	***0.33***	*0.78*	*0.96*		
EWOC	2.5	3.3	26.4	61.9	5.8	0.0	10.9	0
TR	2.5	3.0	18.4	62.9	12.7	0.4	13.1	0
Hybrid ($\alpha_{\min} = 0.10$)	5.0	5.3	15.2	61.4	12.7	0.3	12.7	0
Hybrid ($\alpha_{\min} = 0.25$)	2.5	2.9	16.0	65.5	12.7	0.4	13.2	0
EAT	5.0	2.9	16.2	62.7	12.4	0.8	12.9	0
TDFB ($\alpha_{\min} = 0.10$)	5.1	3.1	16.7	62.1	12.6	0.5	12.9	0
TDFB ($\alpha_{\min} = 0.25$)	2.5	2.9	17.0	64.5	12.8	0.2	13.1	0
**Scenario 6** ($\gamma = 300, \rho_0 = 0.02$)								
Probability of DLT	*0.02*	*0.06*	*0.16*	***0.33***	*0.58*	*0.79*		
EWOC	2.9	6.5	32.4	49.0	8.9	0.3	10.8	0
TR	2.8	4.7	20.6	51.0	18.2	2.6	13.2	0
Hybrid ($\alpha_{\min} = 0.10$)	5.5	4.7	20.0	50.1	17.8	1.9	12.9	0
Hybrid ($\alpha_{\min} = 0.25$)	2.6	4.3	19.9	54.3	17.6	1.5	13.2	0
EAT	5.3	3.8	18.5	51.8	18.1	2.4	13.2	0
TDFB ($\alpha_{\min} = 0.10$)	5.5	4.6	18.9	50.3	18.2	2.5	12.9	0
TDFB ($\alpha_{\min} = 0.25$)	2.8	4.2	20.9	53.4	17.0	1.8	13.3	0
**Scenario 7** ($\gamma = 350, \rho_0 = 0.01$)								
Probability of DLT	*0.01*	*0.03*	*0.07*	*0.16*	***0.33***	*0.56*		
EWOC	2.6	3.3	10.1	33.1	41.9	9.0	10.0	0
TR	2.6	3.5	6.4	21.5	44.7	21.3	12.4	1
Hybrid ($\alpha_{\min} = 0.10$)	5.1	5.5	5.1	21.5	40.9	21.8	11.9	0
Hybrid ($\alpha_{\min} = 0.25$)	2.6	2.9	5.2	20.6	48.8	19.9	12.5	0
EAT	5.1	2.8	4.6	21.0	44.1	22.4	12.4	0
TDFB ($\alpha_{\min} = 0.10$)	5.2	3.1	7.3	20.6	43.2	20.8	12.0	0
TDFB ($\alpha_{\min} = 0.25$)	2.6	2.9	5.3	21.8	47.9	19.5	12.5	0
**Scenario 8** ($\gamma = 350, \rho_0 = 0.05$)								
Probability of DLT	*0.06*	*0.09*	*0.15*	*0.23*	***0.33***	*0.46*		
EWOC	3.0	6.4	18.5	35.2	28.6	8.3	10.0	0
TR	3.0	4.7	10.9	21.7	33.3	26.4	12.0	0
Hybrid ($\alpha_{\min} = 0.10$)	5.8	6.8	8.3	22.0	32.1	25.1	11.6	1
Hybrid ($\alpha_{\min} = 0.25$)	2.9	4.3	8.5	24.6	34.5	25.2	12.1	0
EAT	5.6	3.8	8.6	20.7	34.2	27.1	12.1	0
TDFB ($\alpha_{\min} = 0.10$)	5.8	4.6	9.7	21.6	32.6	25.7	11.8	0
TDFB ($\alpha_{\min} = 0.25$)	2.8	4.6	8.3	24.0	34.3	26.0	12.2	0
**Scenario 9** ($\gamma = 400, \rho_0 = 0.001$)								
Probability of DLT	*0.00*	*0.00*	*0.01*	*0.04*	*0.13*	***0.33***		
EWOC	2.5	2.6	5.6	8.4	29.8	51.1	8.6	0
TR	2.5	2.6	5.4	6.9	15.2	67.3	9.9	0
Hybrid ($\alpha_{\min} = 0.10$)	5.0	2.8	3.1	8.6	15.8	64.6	9.7	0
Hybrid ($\alpha_{\min} = 0.25$)	2.5	2.6	3.0	6.4	16.9	68.6	10.0	0
EAT	5.0	2.6	2.8	4.1	17.0	68.4	10.1	0
TDFB ($\alpha_{\min} = 0.10$)	5.0	2.6	5.3	4.5	15.4	67.1	9.9	0
TDFB ($\alpha_{\min} = 0.25$)	2.5	2.6	2.9	5.0	18.9	68.1	10.4	0
**Scenario 10** ($\gamma = 400, \rho_0 = 0.03$)								
Probability of DLT	*0.03*	*0.06*	*0.09*	*0.15*	*0.23*	***0.33***		
EWOC	2.7	3.7	7.8	21.7	30.9	33.1	8.9	0
TR	2.7	3.7	5.8	11.7	20.7	55.3	10.2	0
Hybrid ($\alpha_{\min} = 0.10$)	5.8	6.0	6.7	10.2	19.6	51.8	9.7	1
Hybrid ($\alpha_{\min} = 0.25$)	2.7	3.2	5.0	11.0	21.7	56.4	10.2	0
EAT	5.4	3.3	3.8	9.2	19.5	58.8	10.2	0
TDFB ($\alpha_{\min} = 0.10$)	5.5	3.4	7.0	9.7	18.7	55.6	10.0	0
TDFB ($\alpha_{\min} = 0.25$)	2.7	3.1	4.8	11.0	22.1	56.0	10.4	0

Table S3: Experimentation percentages for discrete dose scenarios (TTL $\theta = 0.33$). Denominators (number of times feasibility bound increased across 1000 trials) for approaches not guaranteeing coherence: TR design, 5000; Hybrid design, 19000.

Scenario/ Method	Probability of DLT						Mean Bias	RMSE
	d_1	d_2	d_3	d_4	d_5	d_6		
**Scenario 1** ($\gamma = 165, \rho_0 = 0.25$)								
Probability of DLT	***0.28***	*0.47*	*0.66*	*0.82*	*0.91*	*0.96*		
EWOC (median)	38.0	57.9	3.8	0.2	0.1	0.0	33.3	43.7
EWOC (next dose)	80.2	19.5	0.2	0.1	0.0	0.0	10.1	23.0
TR	60.3	39.3	0.4	0.0	0.0	0.0	20.1	32.0
Hybrid ($\alpha_{\min} = 0.10$)	54.5	44.5	1.0	0.0	0.0	0.0	23.3	34.8
Hybrid ($\alpha_{\min} = 0.25$)	56.7	42.4	0.9	0.0	0.0	0.0	22.1	33.9
EAT	55.6	43.5	0.9	0.0	0.0	0.0	22.7	34.3
TDFB ($\alpha_{\min} = 0.10$)	55.3	43.4	1.3	0.0	0.0	0.0	23.0	34.9
TDFB ($\alpha_{\min} = 0.25$)	57.6	41.0	1.4	0.0	0.0	0.0	21.9	34.1
**Scenario 2** ($\gamma = 175, \rho_0 = 0.30$)								
Probability of DLT	***0.31***	*0.36*	*0.41*	*0.46*	*0.52*	*0.57*		
EWOC (median)	26.6	36.9	23.5	10.7	2.0	0.3	62.8	81.7
EWOC (next dose)	48.6	32.1	16.1	2.7	0.5	0.0	37.2	56.8
TR	22.1	34.3	30.4	10.9	2.0	0.3	68.7	85.7
Hybrid ($\alpha_{\min} = 0.10$)	20.1	33.6	30.2	12.4	2.5	1.2	73.6	91.7
Hybrid ($\alpha_{\min} = 0.25$)	20.6	36.1	29.2	11.7	2.4	0.0	69.6	86.1
EAT	20.8	35.6	27.7	13.4	2.5	0.0	70.6	87.6
TDFB ($\alpha_{\min} = 0.10$)	27.7	32.9	26.8	10.0	2.3	0.3	63.6	82.8
TDFB ($\alpha_{\min} = 0.25$)	20.6	35.1	29.0	12.3	2.7	0.3	71.2	88.4
**Scenario 3** ($\gamma = 200, \rho_0 = 0.03$)								
Probability of DLT	*0.05*	***0.33***	*0.84*	*0.98*	*1.00*	*1.00*		
EWOC (median)	0.0	69.6	30.3	0.1	0.0	0.0	15.3	27.7
EWOC (next dose)	10.7	88.2	1.1	0.0	0.0	0.0	-4.8	17.2
TR	5.0	91.7	3.3	0.0	0.0	0.0	-0.9	14.4
Hybrid ($\alpha_{\min} = 0.10$)	3.6	93.7	2.7	0.0	0.0	0.0	-0.5	12.6
Hybrid ($\alpha_{\min} = 0.25$)	3.8	94.1	2.1	0.0	0.0	0.0	-0.9	12.1
EAT	4.0	92.2	3.8	0.0	0.0	0.0	-0.1	14.0
TDFB ($\alpha_{\min} = 0.10$)	2.7	94.2	3.1	0.0	0.0	0.0	0.2	12.0
TDFB ($\alpha_{\min} = 0.25$)	3.0	94.7	2.3	0.0	0.0	0.0	-0.4	11.5
**Scenario 4** ($\gamma = 250, \rho_0 = 0.05$)								
Probability of DLT	*0.06*	*0.15*	***0.33***	*0.58*	*0.79*	*0.92*		
EWOC (median)	0.0	1.3	54.8	41.7	2.1	0.1	22.5	36.2
EWOC (next dose)	0.0	23.2	71.3	5.5	0.0	0.0	-8.9	26.8
TR	0.0	10.8	77.7	11.3	0.2	0.0	0.5	23.9
Hybrid ($\alpha_{\min} = 0.10$)	0.0	8.4	77.7	13.7	0.2	0.0	2.9	23.9
Hybrid ($\alpha_{\min} = 0.25$)	0.0	11.2	77.7	11.0	0.1	0.0	0.0	23.8
EAT	0.0	11.7	79.8	8.4	0.1	0.0	-1.6	22.6
TDFB ($\alpha_{\min} = 0.10$)	0.0	9.1	80.9	9.9	0.1	0.0	0.5	22.0
TDFB ($\alpha_{\min} = 0.25$)	0.0	11.2	77.3	11.5	0.0	0.0	0.2	23.8
**Scenario 5** ($\gamma = 300, \rho_0 = 0.001$)								
Probability of DLT	*0.00*	*0.01*	*0.07*	***0.33***	*0.78*	*0.96*		
EWOC (median)	0.0	0.0	0.0	49.8	49.1	1.1	25.7	36.6
EWOC (next dose)	0.0	0.0	17.7	79.4	2.9	0.0	-7.4	22.7
TR	0.0	0.0	11.9	82.7	5.4	0.0	-3.3	20.8
Hybrid ($\alpha_{\min} = 0.10$)	0.0	0.0	10.4	82.9	6.7	0.0	-1.9	20.7
Hybrid ($\alpha_{\min} = 0.25$)	0.0	0.0	10.2	86.0	3.8	0.0	-3.2	18.7
EAT	0.0	0.0	9.9	85.4	4.7	0.0	-2.6	19.1
TDFB ($\alpha_{\min} = 0.10$)	0.0	0.0	10.7	84.0	5.3	0.0	-2.7	20.0
TDFB ($\alpha_{\min} = 0.25$)	0.0	0.0	9.5	84.3	6.2	0.0	-1.7	19.8
**Scenario 6** ($\gamma = 300, \rho_0 = 0.02$)								
Probability of DLT	*0.02*	*0.06*	*0.16*	***0.33***	*0.58*	*0.79*		
EWOC (median)	0.0	0.0	1.4	50.4	44.5	3.7	25.3	39.0
EWOC (next dose)	0.0	0.0	27.6	65.6	6.8	0.0	-10.4	29.3
TR	0.0	0.0	14.7	73.8	11.3	0.2	-1.5	25.9
Hybrid ($\alpha_{\min} = 0.10$)	0.0	0.0	14.2	72.3	13.1	0.4	-0.2	26.9
Hybrid ($\alpha_{\min} = 0.25$)	0.0	0.0	12.5	75.7	11.8	0.0	-0.4	24.7
EAT	0.0	0.0	14.8	74.6	10.4	0.2	-2.0	25.5
TDFB ($\alpha_{\min} = 0.10$)	0.0	0.0	12.6	74.1	13.0	0.3	0.5	25.9
TDFB ($\alpha_{\min} = 0.25$)	0.0	0.0	16.5	72.9	10.4	0.2	-2.9	26.3
**Scenario 7** ($\gamma = 350, \rho_0 = 0.01$)								
Probability of DLT	*0.01*	*0.03*	*0.07*	*0.16*	***0.33***	*0.56*		
EWOC (median)	0.0	0.0	0.0	2.1	44.2	53.7	25.8	37.4
EWOC (next dose)	0.0	0.0	0.3	30.5	61.3	7.9	-11.6	31.5
TR	0.0	0.0	0.0	15.1	70.4	14.5	-0.3	27.2
Hybrid ($\alpha_{\min} = 0.10$)	0.0	0.0	0.0	17.4	64.5	18.1	0.4	29.8
Hybrid ($\alpha_{\min} = 0.25$)	0.0	0.0	0.0	16.5	68.5	15.0	-0.8	28.1
EAT	0.0	0.0	0.3	19.0	65.9	14.8	-2.4	29.6
TDFB ($\alpha_{\min} = 0.10$)	0.0	0.0	0.0	17.2	66.9	15.9	-0.7	28.8
TDFB ($\alpha_{\min} = 0.25$)	0.0	0.0	0.1	18.2	67.8	13.9	-2.3	28.5
**Scenario 8** ($\gamma = 350, \rho_0 = 0.05$)								
Probability of DLT	*0.06*	*0.09*	*0.15*	*0.23*	***0.33***	*0.46*		
EWOC (median)	0.0	0.0	0.9	12.4	40.1	46.6	16.2	39.6
EWOC (next dose)	0.0	0.2	7.9	38.1	43.9	9.9	-22.3	45.1
TR	0.0	0.0	0.5	21.6	49.8	28.1	2.8	36.0
Hybrid ($\alpha_{\min} = 0.10$)	0.0	0.0	1.0	20.5	50.0	28.5	3.0	36.4
Hybrid ($\alpha_{\min} = 0.25$)	0.0	0.0	0.5	22.2	49.0	28.3	2.6	36.2
EAT	0.0	0.0	0.7	21.5	52.0	25.8	1.5	35.4
TDFB ($\alpha_{\min} = 0.10$)	0.0	0.0	0.7	20.1	51.2	28.0	3.3	35.7
TDFB ($\alpha_{\min} = 0.25$)	0.0	0.0	1.1	21.7	53.4	23.8	-0.1	35.3
**Scenario 9** ($\gamma = 400, \rho_0 = 0.001$)								
Probability of DLT	*0.00*	*0.00*	*0.01*	*0.04*	*0.13*	***0.33***		
EWOC (median)	0.0	0.0	0.0	0.0	0.4	99.6	-0.2	3.2
EWOC (next dose)	0.0	0.0	0.0	0.1	24.0	75.9	-12.1	24.7
TR	0.0	0.0	0.0	0.0	9.7	90.3	-4.9	15.6
Hybrid ($\alpha_{\min} = 0.10$)	0.0	0.0	0.0	0.1	12.5	87.4	-6.4	18.0
Hybrid ($\alpha_{\min} = 0.25$)	0.0	0.0	0.0	0.1	8.1	91.8	-4.2	14.6
EAT	0.0	0.0	0.0	0.0	11.1	88.9	-5.6	16.7
TDFB ($\alpha_{\min} = 0.10$)	0.0	0.0	0.0	0.1	10.5	89.4	-5.4	16.5
TDFB ($\alpha_{\min} = 0.25$)	0.0	0.0	0.0	0.1	11.5	88.4	-5.9	17.3
**Scenario 10** ($\gamma = 400, \rho_0 = 0.03$)								
Probability of DLT	*0.03*	*0.06*	*0.09*	*0.15*	*0.23*	***0.33***		
EWOC (median)	0.0	0.0	0.0	0.5	11.2	88.3	-6.1	18.2
EWOC (next dose)	0.0	0.0	0.2	6.8	41.0	52.0	-27.6	41.8
TR	0.0	0.0	0.0	1.4	18.3	80.3	-10.6	24.4
Hybrid ($\alpha_{\min} = 0.10$)	0.0	0.0	0.1	1.1	19.7	79.1	-11.1	25.0
Hybrid ($\alpha_{\min} = 0.25$)	0.0	0.0	0.0	1.3	20.5	78.2	-11.6	25.4
EAT	0.0	0.0	0.0	1.6	19.2	79.2	-11.2	25.3
TDFB ($\alpha_{\min} = 0.10$)	0.0	0.0	0.0	1.4	19.3	79.3	-11.1	25.0
TDFB ($\alpha_{\min} = 0.25$)	0.0	0.0	0.1	1.3	20.9	77.7	-11.9	26.0

Table S4: Recommendation percentages, bias and RMSE for discrete dose scenarios (TTL $\theta = 0.33$). For all approaches except EWOC, posterior median of MTD equals next dose to be given.

Continuous Dose Scenarios				
Scenario	MTD (γ)	ρ_0	$\mathbb{P}$ (DLT at $x_{\max}$)	β
Power	250	0	1	0.143
Hyperbolic Tangent	250	0.13	0.69	1.062

Discrete Dose Scenarios						
Scenario	$\mathbb{P}(DLT)$					
	d_1	d_2	d_3	d_4	d_5	d_6
Power	0.02	0.17	**0.33**	0.51	0.70	0.90
Hyperbolic Tangent	0.15	0.23	**0.33**	0.44	0.55	0.65

Table S5: Dose-toxicity scenarios (for both continuous dose interval and discrete dose settings) for sensitivity analysis. MTDs are shown in bold.

Scenario/ Model	Probability of DLT							Mean No. DLTs	Coherence Violations
	$[0, 0.15]$	$(0.15, 0.25]$	$(0.25, 0.30]$	$(0.30, 0.35]$	$(0.35, 0.40]$	$(0.40, 0.50]$	$(0.50, 1]$		
**Power** ($\gamma = 250$)									
EWOC	9.0	26.5	17.9	16.9	11.7	13.9	4.2	11.8	0
TR	5.8	14.6	13.9	17.2	15.0	21.3	12.2	14.1	14
Hybrid ($\alpha_{\min} = 0.10$)	10.6	11.7	11.4	15.8	14.5	22.4	13.6	13.7	11
Hybrid ($\alpha_{\min} = 0.25$)	5.5	14.4	13.6	16.0	16.7	21.9	11.9	14.1	6
EAT	7.4	11.6	13.8	14.8	15.4	22.1	14.9	14.2	0
TDFB ($\alpha_{\min} = 0.10$)	10.4	12.0	12.2	14.0	16.5	21.6	13.2	13.8	0
TDFB ($\alpha_{\min} = 0.25$)	5.6	15.0	13.9	16.1	16.5	21.3	11.6	14.1	0
**Hyperbolic Tangent** ($\gamma = 250$)									
EWOC	5.0	24.6	23.5	20.2	12.8	11.5	2.5	11.9	0
TR	3.3	10.4	16.0	19.6	17.7	23.7	9.3	14.0	8
Hybrid ($\alpha_{\min} = 0.10$)	7.4	15.1	13.5	17.8	17.4	21.4	7.4	13.6	13
Hybrid ($\alpha_{\min} = 0.25$)	3.1	9.5	16.6	20.6	19.1	23.2	7.9	14.2	7
EAT	7.1	10.5	14.7	17.1	17.8	23.6	9.2	14.0	0
TDFB ($\alpha_{\min} = 0.10$)	7.7	13.0	14.7	17.2	16.3	21.5	9.6	13.6	0
TDFB ($\alpha_{\min} = 0.25$)	3.4	11.7	16.3	18.3	19.8	22.4	8.2	14.1	0

Table S6: Experimentation percentages for scenarios specified under power and hyperbolic tangent models with continuous dose interval (TTL $\theta = 0.33$). Denominators (number of times feasibility bound increased across 1000 trials) for approaches not guaranteeing coherence: TR design, 5000; Hybrid design, 19000.

Scenario/ Model	Probability of DLT [0, 0.15]	(0.15, 0.25]	(0.25, 0.30]	(0.30, 0.35]	(0.35, 0.40]	(0.40, 0.50]	(0.50, 1]	Mean Bias	RMSE
**Power** ($\gamma = 250$)									
EWOC (median)	0.0	2.2	7.6	15.2	20.3	33.8	20.9	24.1	36.7
EWOC (next dose)	1.7	21.7	26.3	22.0	15.3	11.8	1.2	-7.5	24.1
TR	0.1	9.0	19.4	24.3	21.5	21.1	4.6	4.3	23.7
Hybrid ($\alpha_{\min} = 0.10$)	0.2	7.1	15.9	23.6	24.5	24.2	4.5	7.1	23.8
Hybrid ($\alpha_{\min} = 0.25$)	0.2	8.9	18.1	23.9	23.7	21.3	3.9	4.7	23.2
EAT	0.2	8.7	17.1	27.0	20.1	22.3	4.6	4.8	23.1
TDFB ($\alpha_{\min} = 0.10$)	0.1	7.4	17.8	23.9	22.5	23.5	4.8	6.6	24.1
TDFB ($\alpha_{\min} = 0.25$)	0.0	10.6	19.8	23.2	21.3	20.6	4.5	4.1	24.0
**Hyperbolic Tangent** ($\gamma = 250$)									
EWOC (median)	0.1	6.1	14.6	20.4	20.9	29.3	8.6	18.5	44.7
EWOC (next dose)	0.7	22.3	24.5	27.1	14.9	9.5	1.0	-13.8	37.8
TR	0.0	4.6	15.2	25.1	26.3	26.2	2.6	12.5	34.4
Hybrid ($\alpha_{\min} = 0.10$)	0.0	4.3	17.2	25.4	25.6	23.9	3.6	11.8	36.3
Hybrid ($\alpha_{\min} = 0.25$)	0.1	6.1	15.4	25.6	28.0	22.2	2.6	9.7	34.5
EAT	0.0	5.0	15.0	28.3	26.3	22.5	2.9	10.8	34.3
TDFB ($\alpha_{\min} = 0.10$)	0.1	5.0	15.1	25.0	26.5	24.0	4.3	13.1	37.2
TDFB ($\alpha_{\min} = 0.25$)	0.1	5.9	15.8	29.0	24.1	21.7	3.4	9.6	35.1

Table S7: Recommendation percentages, bias and RMSE for scenarios specified under the power and hyperbolic tangent models with continuous dose interval (TTL $\theta = 0.33$). For all approaches except EWOC, posterior median of MTD equals next dose to be given.

Scenario/ Method	Dose Level						Mean No. DLTs	Coherence Violations
	d_1	d_2	d_3	d_4	d_5	d_6		
**Power** ($\gamma = 250$)								
Probability of DLT	*0.02*	*0.17*	***0.33***	*0.51*	*0.70*	*0.90*		
EWOC	4.7	31.2	50.8	12.5	0.8	0.0	11.7	0
TR	3.9	16.9	51.0	24.6	3.4	0.3	14.0	0
Hybrid ($\alpha_{\text{min}} = 0.10$)	6.6	16.6	49.8	23.3	3.5	0.3	13.8	0
Hybrid ($\alpha_{\text{min}} = 0.25$)	3.5	15.7	51.8	25.3	3.6	0.1	14.1	0
EAT	6.4	14.2	51.0	23.5	4.4	0.6	14.0	0
TDFB ($\alpha_{\text{min}} = 0.10$)	6.3	15.2	50.0	24.6	3.6	0.3	13.8	0
TDFB ($\alpha_{\text{min}} = 0.25$)	3.6	17.3	51.6	24.3	3.0	0.3	14.1	0
**Hyperbolic Tangent** ($\gamma = 250$)								
Probability of DLT	*0.15*	*0.23*	***0.33***	*0.44*	*0.55*	*0.65*		
EWOC	9.9	33.6	40.4	14.1	1.9	0.1	11.9	0
TR	6.1	19.2	37.9	27.3	7.7	1.8	14.0	3
Hybrid ($\alpha_{\text{min}} = 0.10$)	10.5	19.0	36.5	25.5	7.2	1.4	13.5	0
Hybrid ($\alpha_{\text{min}} = 0.25$)	5.3	18.5	40.8	26.9	7.2	1.2	14.1	0
EAT	8.4	16.6	37.0	27.3	8.4	2.2	14.1	0
TDFB ($\alpha_{\text{min}} = 0.10$)	10.6	18.9	38.1	23.5	7.3	1.7	13.7	0
TDFB ($\alpha_{\text{min}} = 0.25$)	5.9	19.4	38.6	26.6	7.7	1.8	14.1	0

Table S8: Experimentation percentages for scenarios specified under power and hyperbolic tangent models with discrete doses (TTL $\theta = 0.33$). Denominators (number of times feasibility bound increased across 1000 trials) for approaches not guaranteeing coherence: TR design, 5000; Hybrid design, 19000.

Scenario/ Method	Probability of DLT						Mean Bias	RMSE
	d_1	d_2	d_3	d_4	d_5	d_6		
**Power** ($\gamma = 250$)								
Probability of DLT	*0.02*	*0.17*	***0.33***	*0.51*	*0.70*	*0.90*		
EWOC (median)	0.0	2.5	53.3	40.5	3.5	0.2	22.8	38.3
EWOC (next dose)	0.3	28.3	63.2	8.1	0.1	0.0	-10.3	30.8
TR	0.0	10.9	71.4	17.2	0.5	0.0	3.6	27.4
Hybrid ($\alpha_{\text{min}} = 0.10$)	0.0	9.5	72.1	17.6	0.8	0.0	4.8	27.5
Hybrid ($\alpha_{\text{min}} = 0.25$)	0.0	11.3	70.3	17.9	0.5	0.0	3.8	27.9
EAT	0.0	8.8	75.0	15.8	0.4	0.0	3.9	25.6
TDFB ($\alpha_{\text{min}} = 0.10$)	0.0	7.9	74.9	17.0	0.2	0.0	4.8	25.3
TDFB ($\alpha_{\text{min}} = 0.25$)	0.0	11.1	72.5	15.8	0.6	0.0	3.0	27.1
**Hyperbolic Tangent** ($\gamma = 250$)								
Probability of DLT	*0.15*	*0.23*	***0.33***	*0.44*	*0.55*	*0.65*		
EWOC (median)	0.4	11.2	46.5	34.9	6.2	0.8	18.9	44.6
EWOC (next dose)	3.6	36.0	47.6	12.3	0.5	0.0	-14.9	40.2
TR	0.3	14.7	51.5	29.2	4.3	0.0	11.2	39.5
Hybrid ($\alpha_{\text{min}} = 0.10$)	0.3	14.0	49.2	31.6	4.6	0.3	13.6	41.2
Hybrid ($\alpha_{\text{min}} = 0.25$)	0.3	14.5	56.4	26.4	2.3	0.1	8.1	36.1
EAT	0.4	14.7	52.4	29.4	3.1	0.0	10.1	38.1
TDFB ($\alpha_{\text{min}} = 0.10$)	0.4	14.9	53.3	27.1	4.1	0.2	10.1	39.3
TDFB ($\alpha_{\text{min}} = 0.25$)	0.4	15.6	52.5	28.0	3.0	0.5	9.6	39.3

Table S9: Recommendation percentages, bias and RMSE for scenarios specified under power and hyperbolic tangent models with discrete doses (TTL $\theta = 0.33$). For all approaches except EWOC, posterior median of MTD equals next dose to be given.

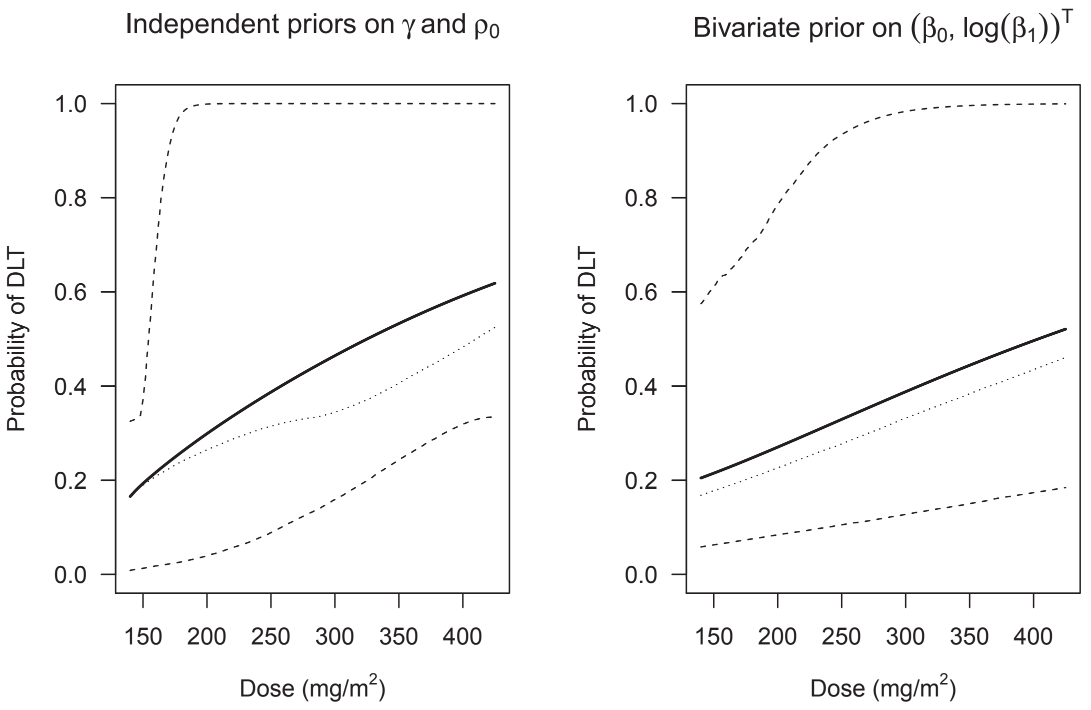

Figure S1: Prior dose-toxicity relationships from uniform priors on γ and ρ_0, and bivariate normal priors on the vector $(\beta_0, \log(\beta_1))$. Lines in plots include mean (solid line), median (dotted) and 95% credibility interval (dashed).

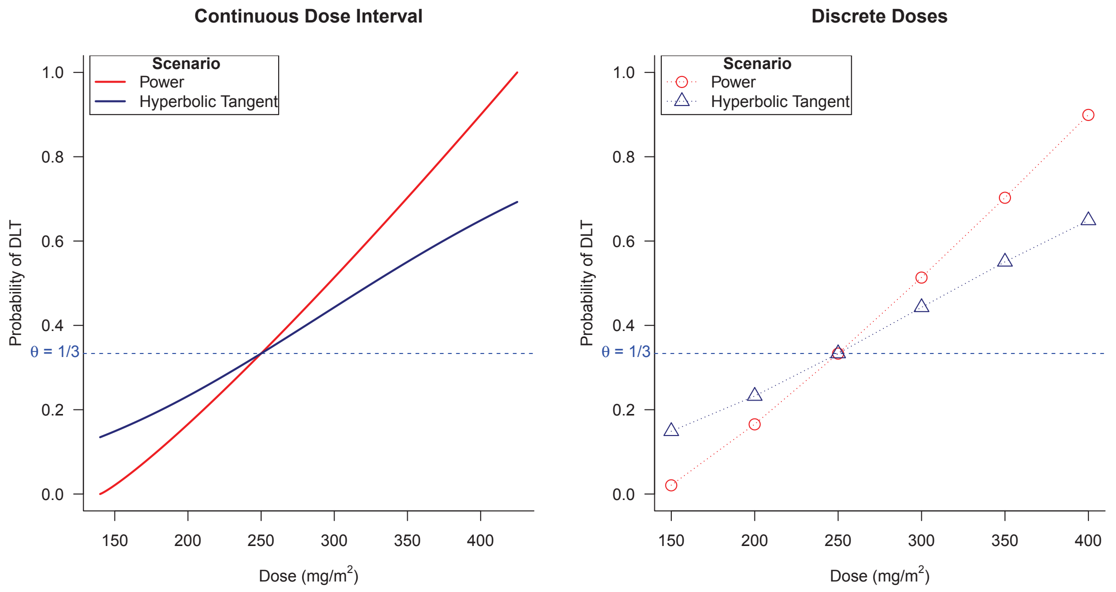

Figure S2: Dose-toxicity scenarios for continuous dose interval (left plot) and discrete doses (right plot) used in sensitivity analysis. TTL $\theta = 0.33$ (blue dashed horizontal line).
